# Structure of the ATP synthase from *Mycobacterium smegmatis* provides targets for treating tuberculosis

**DOI:** 10.1073/pnas.2111899118

**Published:** 2021-11-15

**Authors:** Martin G. Montgomery, Jessica Petri, Tobias E. Spikes, John E. Walker

**Affiliations:** ^a^The Medical Research Council Mitochondrial Biology Unit, University of Cambridge, Cambridge CB2 0XY, United Kingdom

**Keywords:** mycobacteria, ATP synthase, structure, regulation, rotary mechanism

## Abstract

The structure has been determined by electron cryomicroscopy of the adenosine triphosphate (ATP) synthase from *Mycobacterium smegmatis*. This analysis confirms features in a prior description of the structure of the enzyme, but it also describes other highly significant attributes not recognized before that are crucial for understanding the mechanism and regulation of the mycobacterial enzyme. First, we resolved not only the three main states in the catalytic cycle described before but also eight substates that portray structural and mechanistic changes occurring during a 360° catalytic cycle. Second, a mechanism of auto-inhibition of ATP hydrolysis involves not only the engagement of the C-terminal region of an α-subunit in a loop in the γ-subunit, as proposed before, but also a “fail-safe” mechanism involving the b′-subunit in the peripheral stalk that enhances engagement. A third unreported characteristic is that the fused bδ-subunit contains a duplicated domain in its N-terminal region where the two copies of the domain participate in similar modes of attachment of the two of three N-terminal regions of the α-subunits. The auto-inhibitory plus the associated “fail-safe” mechanisms and the modes of attachment of the α-subunits provide targets for development of innovative antitubercular drugs. The structure also provides support for an observation made in the bovine ATP synthase that the transmembrane proton-motive force that provides the energy to drive the rotary mechanism is delivered directly and tangentially to the rotor via a Grotthuss water chain in a polar L-shaped tunnel.

## Significance

As the world tackles the COVID-19 pandemic, other widespread infectious diseases, including tuberculosis (TB), take their toll on humans, and those with TB are more likely to die from COVID-19 infection. Bedaquiline (BD), an anti-TB drug, combats multidrug-resistant *Mycobacterium tuberculosis* by preventing a molecular machine known as the adenosine triphosphate (ATP) synthase from generating ATP, the fuel needed to keep it alive. However, BD-resistant strains of *M. tuberculosis* have arisen. Here, we describe features of the mycobacterial ATP synthase that are not present in the human enzyme. Potentially these features can be exploited for the development of new anti-TB drugs unrelated to BD to prevent and cure TB by inhibiting the formation of ATP by the pathogen.

The adenosine triphosphate (ATP) synthase in *Mycobacterium tuberculosis* is the antimicrobial target for the treatment of tuberculosis (TB) with the drug bedaquiline (BD), but despite the effectiveness of this drug for treatment of multiple extremely and totally drug-resistant strains of the pathogen, BD resistance has been observed already in clinical isolates (1). Moreover, patients treated with BD have a fivefold higher risk of death than a placebo control group, possibly because of its effects on the human ATP synthase (2). Thus, one possible strategy to combat the growing threat of TB is to identify other inhibitors acting at novel sites of the mycobacterial ATP synthase that could be developed into antibiotics without these side effects. Detailed knowledge of the structure and properties of the enzyme would be helpful in this quest, and the closely related ATP synthase in the nonpathogenic species *Mycobacterium smegmatis* provides an excellent model for the enzyme from the pathogen. Like other mycobacteria*, M. smegmatis* is covered by a complex cell envelope of three distinct layers, consisting of the inner plasma membrane (IPM), the peptidoglycan–arabinogalactan complex, and the outer membrane, which is linked covalently to the arabinogalactan (3). The membrane domain of the mycobacterial ATP synthase is embedded in the IPM, with the catalytic F_1_-domain of the enzyme extending into the bacterial cytoplasm and attached to the membrane domain by a central stalk and a peripheral stalk (PS). As do other ATP synthases in eubacteria, chloroplasts, and mitochondria, the enzyme couples a proton-motive force (pmf) across the IPM generated by respiration to the synthesis in the F_1_-domain of the enzyme of ATP from adenosine diphosphate (ADP) and phosphate by a mechanical rotary mechanism (4). The mycobacterial rotor consists of a membrane-bound ring of nine c-subunits (5), attached to an elongated central stalk, made of single copies of the γ- and ε-subunits. This central stalk is common to all F-type ATP synthases and penetrates into the spherical part of the F_1_-domain, an assembly of three α-subunits and three β-subunits arranged in alternation around the central axis (6). The turning of the asymmetrical rotor brings about a series of conformational changes in the three catalytic sites, which lie at three of the six interfaces between α- and β-subunits, leading sequentially to the binding of the substrates ADP and phosphate, the formation of ATP, and finally the release of ATP. Three ATP molecules are produced by each 360° rotary cycle (4). The α_3_β_3_-domain is attached to the a-subunit in the membrane domain by the PS (7). The mycobacterial PS is made of two proteins, bδ and b′. The bδ-subunit has been described as a covalent fusion of the separate b- and δ-subunits found in many other eubacteria (8). The b′-subunit is orthologous (but not identical) to the b-component of the bδ-subunit, and both the b-component and the b′-subunit have N-terminal hydrophobic regions, each capable of forming single transmembrane α-helices. In other bacterial ATP synthases of known structure (9–11), the corresponding α-helices interact with the final component of the stator, the single a-subunit, and hold it in contact with the rotating c-ring in order to maintain the integrity of the two proton half-channels, thereby maintaining the coupling of ATP synthases to the transmembrane pmf. Protons cross the membrane via two half-channels at the interface between the rotating ring and the relatively static stator component of the ATP synthase (4, 12, 13), and they provide the energy from the pmf required to generate the turning of the rotor. Previously, we have described the atomic structure of the F_1_-catalytic domain of the ATP synthase from *M. smegmatis* determined by X-ray crystallography (6), and recently an independent structure has been described of the entire mycobacterial complex determined by electron cryomicroscopy (cryo-EM) of purified single particles of the enzyme inhibited by BD (14). In this analysis, three catalytic states of the enzyme were resolved corresponding to the same inhibited catalytic state in the three different positions relative to the single PS. In addition, a novel method of auto-inhibition was suggested by the association of one of the extended structures in the C-terminal region of the α-subunits with a loop region in the γ-subunit. Both the C-terminal extension in the α-subunits and the additional loop in the γ-subunit are conserved in mycobacterial ATP synthases but are not found elsewhere among eubacterial, chloroplast, or mitochondrial ATP synthases.

Here, we describe an independent structural analysis of the mycobacterial enzyme inhibited by BD. This structure not only confirms many of the features in the earlier structures of the enzyme (6, 14) but also it provides evidence of other highly significant features that have not been described before that are crucial for understanding the mechanism and regulation of the mycobacterial enzyme. Most notably, we have resolved not only the three main states in the catalytic cycle but also eight additional substates, thereby elaborating structural and mechanistic changes that occur during a 360° catalytic cycle. Second, the auto-inhibitory mechanism has been extended to involve not only the C-terminal region of an α-subunit and a loop in the γ-subunit, as proposed before (14), but also the b′-subunit in the PS. Third, we have demonstrated that the fused bδ-subunit contains a duplicated domain in its N-terminal region where the two copies of the domain participate in similar modes of attachment of the two of three N-terminal regions of the α-subunits. Another significant feature of the structure is that it provides further support for an observation made in the bovine ATP synthase that the transmembrane pmf that provides the energy to turn the enzyme’s rotor is delivered directly and tangentially to the rotor via a Grotthuss water chain in a polar L-shaped tunnel formed at the inlet half-channel (15). Subsequently, a similar mechanism has been proposed to operate in the yeast V-type ATPase, based on a structure determined at *ca.* 2.7 Å, where ordered chains of water molecules were observed in both the inlet and outlet half-channels (16)

## Results and Discussion

### The Structure of Mycobacterial ATP Synthase.

**Fig. 1.**
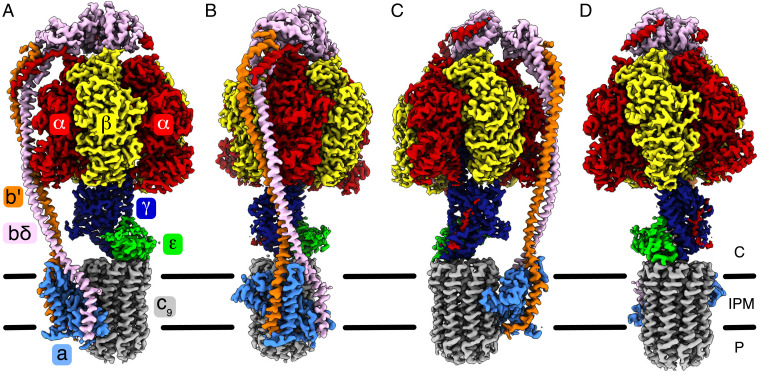
The structure and subunit composition of ATP synthase from *M. smegmatis*. (*A*–*D*) Four views of the electron density of the intact enzyme in state s1a (EMD-12377). The colors corresponding to each subunit are indicated in *A*. In *B*–*D*, the views are rotated by 90° from left to right about the vertical axis running through the central stalk and the center of the c_9_-ring. The mycobacterial ATP synthase consists of three copies of the α-subunit, with an extended C terminus unique to *Mycobacteria*; three copies of the β-subunit; nine copies of the c-subunit; and one copy each of the γ-, ε-, a-, b′-, and bδ-subunits. The latter is also unique to *Mycobacteria* and comprises a canonical b-subunit fused to a canonical δ-subunit via an additional bundle of seven α-helices. The black bars represent the IPM. Protons are translocated from the periplasm (P) to the cytoplasm via the interface between a- and c-subunits.

Single particles of the ATP synthase from *M. smegmatis* purified in the presence of detergent (*SI Appendix*, Fig. S1 and Table S1) and inhibited with BD were subject to cryo-EM analysis. A total of 63 maps and 27 models of the enzyme were resolved and refined with resolutions ranging from 2.11 to 4.15 Å (*SI Appendix*, Figs. S2 and S3 and Tables S2 and S3). The best-resolved model, that of substate S1a (see below; Protein Data Bank [PDB] ID code 7NJK) is at a resolution of 2.5 to 3.4 Å. For the quality of the electron density of each subunit, see *SI Appendix*, Fig. S4. The model of substate S1a (Fig. 1 and Movie S1) contains the following residues: α_E_ 5 to 21, 29 to 406, 411 to 521; α_TP_ 5 to 21, 29 to 406, 411 to 521; α_DP_ 6 to 406, 413 to 521, 527 to 545; β_E_ 8 to 471; β_TP_ 7 to 475; β_DP_ 8 to 475; γ 3 to 213, 220 to 304; ε 3 to 120; a 10 to 247; bδ 1 to 162, 169 to 444; b′ 22 to 166; c 3 to 86 (in all nine copies). Each nucleotide binding site in the three α-subunits and in subunit β_TP_ is occupied by an ATP molecule with an accompanying Mg^2+^ ion, while the site in subunit β_DP_ has ADP and an accompanying Mg^2+^ ion. The sixth nucleotide binding site in subunit β_E_ contains an ADP molecule with neither accompanying Mg^2+^ nor phosphate (see *SI Appendix*, Fig. S5). For comparison, in a similar cryo-EM analysis of single particles of the same mycobacterial ATP synthase, 12 maps and models were resolved with resolutions ranging from 3.2 to 3.7 Å (14). In that study, the best-resolved model (PDB ID code 7JGA, 3.2 Å) has the following residues: α_E_ 7 to 520; α_TP_ 9 to 22, 29 to 515, 533 to 546; α_DP_ 8 to 22, 28 to 545; β_E_ 8 to 471; β_TP_ 8 to 471; β_DP_ 8 to 471; γ 4 to 164, 178 to 213, 220 to 303; ε 3 to 119; a 31 to 113, 123 to 246; bδ 1 to 157, 169 to 331, 337 to 444; b′ 24 to 164; c 5 to 85 (in all nine copies). The occupancy of nucleotides is as follows (PDB ID code 7JGA): ATP is present in all three α-subunits, and ADP is present in β_TP_, with a Mg^2+^ ion coordinated by the nucleotide and a threonine residue (αThr179 or βThr167) and water molecules in each case. Neither β_DP_ nor β_E_ has a bound nucleotide, probably as there were no exogenous nucleotides present throughout the purification process. A phosphate has been modeled in β_E_ at the top of the binding site near to Glu196, Asp254, and Arg376. One major difference is that in the prior study (14) BD was well-resolved bound to the c_9_-ring at the interface with the a-subunit. In the current study, the concentration of BD employed was almost an order of magnitude lower (25 μM instead of 200 μM), and this lower concentration proved to be suboptimal for the detection of the BD itself, as the drug was present in only a minority of particles. Therefore, in the various states and substates (see below) the density for BD is highly variable. Also, in the earlier study, a different detergent was used in the isolation of the intact particles of the enzyme, and that also may have influenced the binding of BD to the enzyme. While it is possible that differing detergent, BD, or nucleotide concentrations in the enzyme preparations may be responsible for the wider range of substates observed, each of the substates in the current work have exactly the same nucleotide occupancies and this is mirrored in recently reported substates in the *Escherichia coli* enzyme (17), suggesting that the resolved substates are nucleotide-independent. It is also possible that further refinement of the particle classes in the previous data (14) could reveal some or all of the substates presented here. However, in the current work, by combining the membrane domains of all of the particles, bound BD could be modeled in the same position as previously reported in close proximity to the N-terminal region of aH5 of the c-subunit in a position that would inhibit ATP synthesis (see *SI Appendix*, Fig. S6). This position is the “lagging” site (14). In some reconstructions, partial nonprotein densities were observed at the opposite side of the a–c interface in close proximity to the C-terminal region of aH5 as in the prior publication. However, the densities were poorly defined and it would be tenuous, in the absence of previously published results (14), to assign them definitively as BD molecules. Similar to the prior study (14), the electron density maps for each of states S1 to S3 has density for both tethered and untethered forms, and hence those particle classes contain enzymes that are auto-inhibited and others that are not inhibited by this mechanism. In the tethered forms the mycobacterial-specific extensions of the α-subunit make contact with the γ-subunit. In the untethered forms, these regions are disordered in the reconstructions and are presumably freely moving in the adjacent aqueous environment.

### Catalytic States and Substates.

**Fig. 2.**
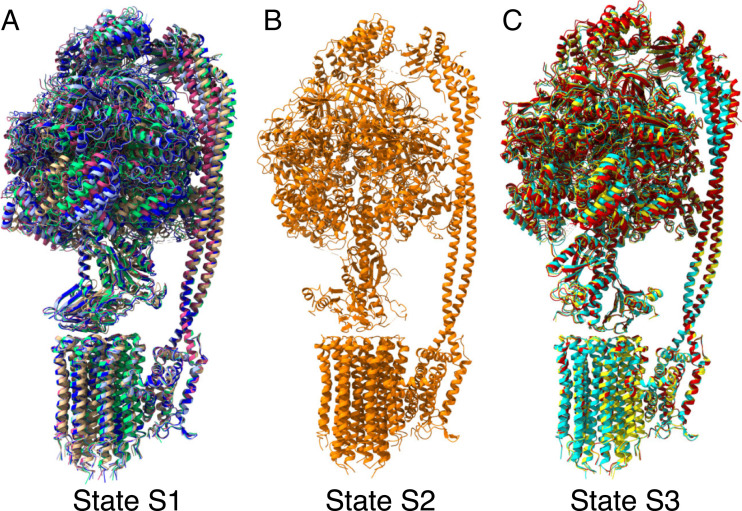
Structures of states and substates in the catalytic cycle of the ATP synthase from *M. smegmatis*. (*A–C*) The structures of the three main states, S1 to S3, defined by the position of the rotor relative to the stator. Each 360° rotary cycle is composed of the transitions S1 to S2, S2 to S3, and S3 to S1. In state S2, the rotor has turned 120° relative to state S1 and in state S3 by 120° relative to state S2. In states S1 and S3, substates S1a to S1e and S3a to S3c, respectively, were resolved. (*A*) Superimposition of substates S1a to S1e, colored tan, green, magenta, pale blue, and dark blue, respectively. (*B*) State S2. (*C*) Superimposition of substates S3a to S3c, colored yellow, cyan, and red, respectively. The substates are aligned via their a-subunits. In states S1 and S3, the F_1_ and PS domains occupy a variety of positions (although in an alignment of the substates via the crown of the F_1_-domain, the nucleotide occupancies of the F_1_ domains are identical and the position of the γ-subunit is approximately constant). In substate S1a, the rotor has turned one c-subunit onward in a hydrolytic direction compared to S1b to S1e (see Fig. 3).

The three catalytic sites in the F_1_ domain of ATP synthases are arranged at 120° intervals around the rotor, and active ATP synthases have been defined in three main states distinguished from each other by the position of the rotor relative to the stator. Extensive classification of the data for the mycobacterial ATP synthase revealed the presence of not only the three main states referred to as S1, S2, and S3 but also of substates within them (Fig. 2). Substates S1a to S1e are in S1, and S3a to S3c are in S3. Two further substates were detected in state S1, and one in state S2, but the number of particles collected in each instance was insufficient to allow accurate models to be built (*SI Appendix*, Fig. S3). Alignment of the substates in S1 and S3 with the three states observed previously via their a-subunits showed that substate S1a (PDB ID code 7NJK) closely matches the earlier state 1 (PDB ID code 7JG5) and S2 (PDB ID code 7NJP) is closely similar to the previous state 2 (PDB ID code 7JG6). In their membrane domains, substates S3a (PDB ID code 7NJQ), S3b (PDB ID code 7NJR), and S3c (PDB ID code 7NJS) are similar to the earlier state 3 (PDB ID code 7JG7), but there are significant deviations in the F_1_ and PS domains (*SI Appendix*, Fig. S7).

The main differences between the substates within each state are that the positions of the PS and F_1_ domains vary such that the F_1_-domain (including the α-helical coiled-coil of the γ-subunit) appears to pivot around the PS during the catalytic cycle (Movies S2 and S3). Pivot points are located around residues 59 to 66 in the b-subunit, residues 34 to 41 in the bδ-subunit, and residues 43 and 233 in the γ-subunit (*SI Appendix*, Figs. S8 and S9). However, alignment of the substates via the “crown” in the F_1_-domain shows that they have identical nucleotide occupancies and that the position of the γ-subunit is also maintained. Alignment of the substates via the a-subunit demonstrates that the c_9_-rings and the membrane α-helices of the a-, b-, and bδ-subunits of the substates are in identical positions, as are the ε-subunits, and in some instances the lower part of the γ-subunit.

**Fig. 3.**
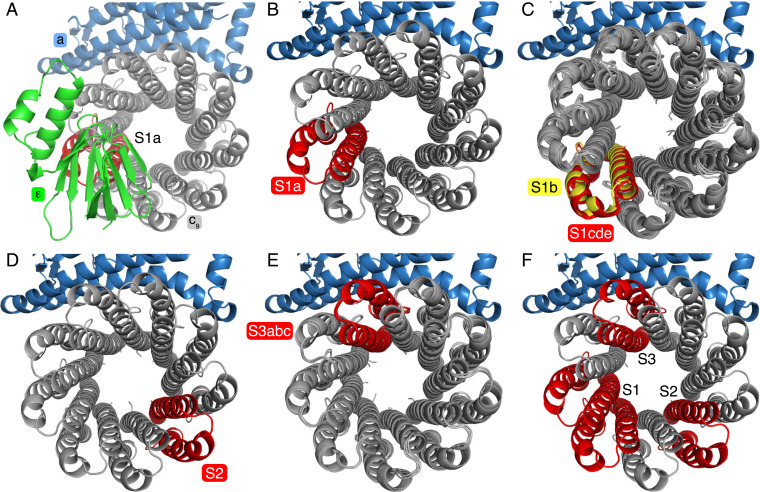
Symmetry mismatch in the substate rotors. By aligning all of the substate models via their a-subunits, the rotation of the c_9_-ring was tracked through the substates via the c-subunit located directly beneath the β-sheet domain of the ε-subunit. (*A*) View from above substate S1a with the ε-, a-, and c-subunits colored green, sky blue, and gray, respectively, with the c-subunit directly beneath the ε-subunit in red. (*B–E*) Similar views of substates with the ε-subunit removed and the equivalent c-subunit in each substate colored. (*B*) Substate S1a. (*C*) Overlays of the c-rings from substates S1b to e. In these substates, the equivalent c-subunits have rotated approximately one c-monomer further round in the synthesis direction (anticlockwise). In *C*, the highlighted c-subunit of S1b (yellow) has rotated by *ca.* 40°, whereas in substates S1c to S1e it has rotated by *ca.* 42° to approximately the same position shown in red. In *D*, state S2 has rotated two c-monomers further round with a corresponding rotation of *ca*. 120°. In *E*, substates S3a to c have rotated by a further three c-monomers (*ca.* 280°). (*F*) Summary of the positions of the c-rings in the three main states S1, S2, and S3.

As in all other F-ATPases, the F_1_ catalytic domain has threefold pseudosymmetry with each catalytic interface of the α- and β-subunits separated by 120°. However, similar to the F-ATPases from *Paracoccus denitrificans* and *Spirulina platensis* which, respectively, have c_12_ and c_15_ rotor rings (9, 18), the mycobacterial c_9_-ring has a symmetry related to that of the F_1_-domain, whereas in other species where the number of c-subunits is not divisible by 3 there is a “symmetry mismatch” between the catalytic and c-ring rotor domains. This symmetry mismatch has been invoked as being part of a mechanism for storing energy transiently in the γ-subunit as the ring rotates (4). This energy is released subsequently in quanta to generate the observed stepping of the rotor (the γ-subunit) in the F_1_-region. Despite the symmetry correspondence between its F_1_ and c-ring domains in the structure of the enzyme from *M. smegmatis*, a symmetry mismatch between the two domains persists here also. The presence of this mismatch is demonstrated by catalytic states S1 to S3, which do not display threefold symmetry and are not separated from each other by 120° (Fig. 3). Thus, as the structures of γ-subunits are highly conserved, it appears that there is a conservation of an energetic mismatch between the two domains that is independent of symmetry. Thus, as illustrated in Fig. 3 and Movie S4, by using the c-subunit directly under the ε-subunit as a marker, the rotation of the ring can be tracked through the substates (Fig. 3*A*). In S1, the rotor turns almost one c-subunit onward (*ca.* 40°) from S1a (Fig. 3*B*), in the direction of synthesis (anticlockwise from above) to substate S1b, which also moves the γ- and ε-subunits relative to the other states. Further round from S1b by *ca.* 2° lie S1c to S1e, which have similar c-ring positions to each other (Fig. 3*C*), but there are large differences in the F_1_ domain and in the PS. The positions of the top of the PS suggest that the order of procession is S1a > S1b > S1e > S1d > S1c. As the inhibitory tether is attached in some of the particles, this movement of the rotor in S1a and S1b may also be responsible for additional differences in the F_1_-domain and the PS compared to S1c to S1e. In S2 (where there are no resolved substates) the c-ring has traveled two c-monomers further round (*ca.*120°), and in state 3 substates S3a to S3c have all traveled on to approximately the same position, four c-monomers (or *ca.* 280°) further on (Fig. 3 *D* and *E*). Again, the position of the PS suggests that the order of substates is either S3c > S3a > S3b or S3b > S3a > S3c. Thus, the order of all the resolved substates in the synthesis direction is either S1a > S1b > S1e > S1d > S1c > S2 > S3c > S3a > S3b (Movie S2) or S1a > S1b > S1e > S1d > S1c > S2 > S3b > S3a > S3c (Movies S3. Among the substates reported in the ATP synthase from *E. coli* (17), one substate, S1a, also corresponds to a rotor turn of one c-subunit. However, relative to the mycobacterial enzyme, the accompanying movement in the γ- and ε-subunits is much less, probably because the *E. coli* ε-subunit is in the inhibitory “up” position (19) where its C-terminal domain occupies the cavity next to the α-helical coiled-coil of the γ-subunit in F_1_-domain of the enzyme, and similarly for the ATP synthase from *Geobacillus stearothermophilus* (11). In the mycobacterial ATP synthase it is not known whether the ε-subunit, which is observed in the “down” position in the structure, can also assume the “up” position, but there is no evidence that it does so (20, 21). Thus, it appears that the mycobacterial PS, as proposed in other ATP synthases where there is a mismatch of symmetries between F_1_ and c-ring domains (4, 22), also acts as a spring that stores and then releases energy to generate the steps in the F_1_-domain during rotation (17, 23) (see *SI Appendix*, Figs. S10 and S11 and Movies S2, S3, and S5, illustrating the transitions during synthesis, in Movies S2 and S3, and in hydrolysis, in Movie S5, by the ATP synthases from *M. smegmatis*).

### Auto-Inhibition of ATP Hydrolysis.

**Fig. 4.**
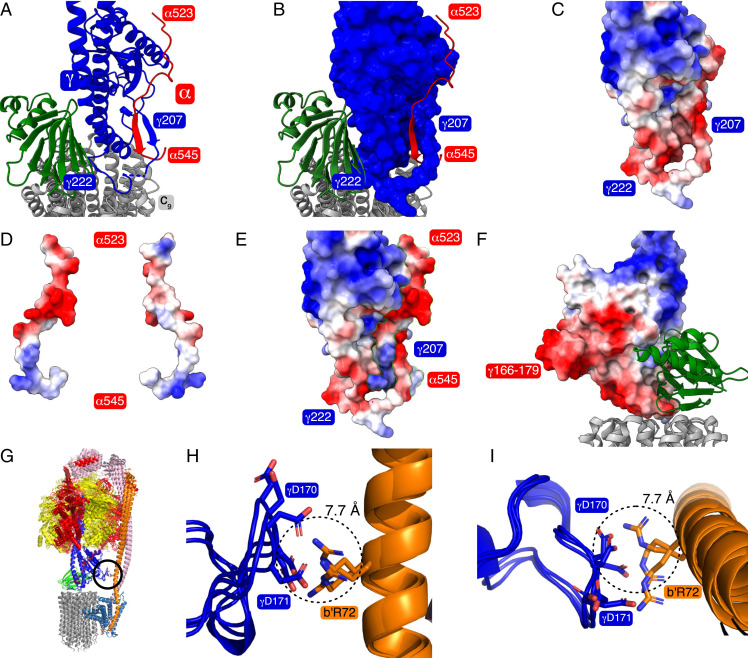
Auto-inhibition of hydrolysis in the ATP synthase from *M. smegmatis*. (*A* and *B*) The extended “hook” provided by residues in the C-terminal region of an α-subunit (red) engaged in a “loop” provided by residues 205 to 227 of a γ-subunit (blue). The ε-subunit (green) is bound to this region of the γ-subunit and attaches the central stalk (subunits γ and ε) to the c_9_-ring (gray). Together, the γ- and ε-subunits plus the c_9_-ring constitute the enzyme's rotor. The subunits are depicted in cartoon representation, except for the γ-subunit in *B*, which is in space-filling format. (*C–E*) The electrostatic potentials of the surfaces of the elements of the hook and loop mechanism of inhibition of ATP hydrolysis. (*C*) The loop. (*D*) The two opposing surfaces of the “hook” with the positively charged surface that engages the negatively charged “loop” during ATP hydrolysis on the left. (*E*) The hook engaged in the loop. (*F*) During ATP synthesis, the positively charged face shown on the right is presented to the positively charged external surface of the “loop” and is repelled. Hence, the inhibitory mechanism is unidirectional. In *A*–*F*, the model of the rotor in state 3 is depicted (PDB ID code 7NKO). (*G*–*L*) The “fail-safe” mechanism augmenting the “hook–loop” catch mechanism involving an interaction between residues γ166 and 179 and b′Arg72. (*G*) The encircled region denotes the “fail-safe” device. It occurs between the rotor and the stator in state 1, and especially in substates S1c, S1d, and S1e, which are shown in cartoon format superimposed via their a-subunits. (*H* and *I*) Close-up views from the side and from above, respectively, showing in stick representation the interaction between negatively charged residues γAsp170 and γAsp171 with positively charged b′Arg72. The diameter of the dotted circle is 7.7 Å corresponding to the distance between the Cα atoms of γAsp171 and bArg72 in substate S1d.

The structure of the ATP synthase from *M. smegmatis* suggests a tether mechanism for the inhibition of ATP hydrolysis, and of the accompanying reversal of the direction of turning of the rotor and expulsion of protons from the bacterial cytoplasm that would happen when the pmf is disrupted, for example under anaerobic conditions (14). This mechanism has two elements based upon structural features that are unique to the mycobacterial ATP synthases. In the first element (Fig. 4 *A*–*F* and *SI Appendix*, Fig. S12) described before (14), residues 520 to 548 extend from the C-terminal region of α-subunits and provide a “hook” which catches into a “loop” provided by residues 212 to 220 in the lower globular domain of the γ-subunit, thereby tethering the stator and rotor together and inhibiting rotation (see also Movie S6). An important feature of this tether mechanism is that it is unidirectional, operating only in the hydrolytic direction of rotation when their interacting surfaces have complementary charges (Fig. 4 *C–E*). In contrast, in the synthetic direction, as the interacting surfaces are both negatively charged they will repel each other, thereby preventing an inhibited complex from forming. This part of the tether mechanism is consistent with the observation that the treatment of the F_1_-ATPase from *M. smegmatis* with trypsin activates its ATP hydrolytic activity as trypsinolysis removes residues 543 to 548 of the α-subunits and disrupts the loop region by cleaving the γ-subunit after residue 219 (6) (see *SI Appendix*, Fig. S13). The second element of this inhibitory mechanism depicted in Fig. 4 *G*–*I* and *SI Appendix*, Figs. S14 and S15 has not been described previously, and it provides a “fail-safe” device. During rotation through S1 in either direction, the γ-subunit approaches very close to the PS at b′Arg72, and potentially a salt bridge could form between this residue and either γAsp170 or γAsp171. Unlike the tether, which can be formed in any of S1 to S3, this feature can form only in S1, when, during rotation, the γ-subunit comes sufficiently close to the PS for a salt bridge to form. Hence, if the hook fails to engage in the loop and form the tether in S2 and S3, in S1 the proximity of b′Arg72 and γAsp170 plus γAsp171, and the formation of the salt bridge, will increase the likelihood of the hook’s engaging in the loop in this state as the hook would be prevented from slipping around the γ-subunit because of the proximity of residues γ168 to 175 to the PS. In *M. tuberculosis*, residues 168 to 175 EGDDAGAD in *M. smegmatis* are replaced by residues 168 to 174 GEDQRSD, and b′Arg72 in *M. smegmatis* is substituted by b′Lys72 in *M. tuberculosis*. Although the changed sequences in *M. tuberculosis* could conceivably affect the fold of the loop and the possibility of forming a salt bridge with b′Lys72, it is highly likely that this region of the γ-subunit would still come into contact with the PS and that the “fail-safe” mechanism will operate in the pathogen also. These observations expand on preliminary biochemical data that residues γ 168 to 175 are involved in regulation of the enzyme (24). These features are not present in the ATP synthases from either *E. coli* (see *SI Appendix*, Fig. S15) or *G. stearothermophilus* where, during a 360° rotary cycle, the γ-subunits do not come close to the PS. In the prior structure of the ATP synthase from *M. smegmatis*, residues 164 to 177 were disordered.

### The PS.

**Fig. 5.**
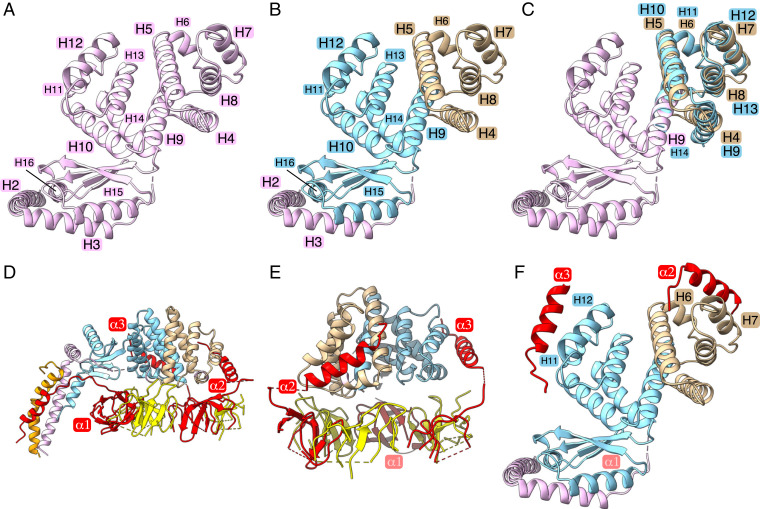
Structure of the bδ-subunit and its interactions with α-subunits in the ATP synthase from *M. smegmatis.* (*A*) The bδ-subunit (pink) viewed from above the crown of the F_1_-domain toward the IPM consists of 16 α-helices, bδH1 to bδH16, with a single intervening β-strand between bδH14 and bδH15 and three additional β-strands between bδH15 and bδH16. In this view, bδH1, which traverses the IPM, is not visible. (*B*) The three domains of the bδ-subunit, with the N-terminal “b”-domain (pink) consisting of bδH1 to bδH3, followed by the central “linking domain” (tan) consisting of bδH4 to bδH8, and the C-terminal “δ”-domain (pale blue), consisting of bδH9 to bδH16. (*C*) Superimposition of the “δ-domain” (pale blue) and the “linking domain” (tan) by a clockwise rotation of *ca.*120° (see Movie S7). (*D* and *E*) Interactions of the N-terminal α-helical regions (labeled α1, α2, and α3) of the three α-subunits (red) with the bδ-subunit. Elements of the α- and β-subunits in the “crown” region of the F_1_-domain are colored red and yellow, respectively. In *D* part of the b-subunit (residues 130 to 166) is orange. In *E* relative to *D*, the view has been rotated by 90° toward the viewer. (*F*) The same view as in *A–C* illustrating the similarity of the modes of binding of N-terminal regions α2 and α3 to the “linking domain” (tan) and the “δ-domain” (pale blue), respectively. In *D*, the N-terminal region α1 is bound close to the PS and interacting with bδH2, bδH16, the immediately preceding β-strand, and the b′-subunit (orange). This mode of interaction differs from those of α2 and α3. For the mode of binding of α2 in other bacterial enzymes see *SI Appendix*, Fig. S20.

The PS is a key component of the enzyme’s stator joining the external surface of the α_3_β_3_-spherical component of the catalytic domain to the a-subunit in the membrane domain, thereby helping to ensure the maintenance of the contact between the membrane domains of the rotor and the stator during catalysis (4). The simplest examples are found in some eubacteria and chloroplasts where the N-terminal domain of the δ-subunit is attached noncovalently to N-terminal extensions of the three α-subunits, and its C-terminal domain forms other noncovalent interactions with the C-terminal regions of two identical b-subunits (as in *E. coli*, for example) or two nonidentical but orthologous b- and b′-subunits, as in cyanobacteria (25, 26) and chloroplasts (27), for example. Metazoan and fungal mitochondrial ATP synthases have a related but more complex PS where the OSCP (oligomycin sensitivity conferral protein) subunit, which is orthologous to the bacterial δ-subunits (28), also binds to the N-terminal regions of the three α-subunits and joins to a long and complex α-helical structure that extends to and penetrates the inner mitochondrial membrane, where it helps to hold the a-subunit of the stator against the c-ring of the rotor. The composition and structure of the PS in mycobacteria has similarities to the PS regions in other prokaryotic ATP synthases, but there are significant differences. In the structure of the *M. smegmatis* ATP synthase, the PS is a complex of a single b′-subunit (similar to b′-subunits in bacteria [see *SI Appendix*, Fig. S16] and chloroplasts) and the unique bδ-subunit. This bδ-subunit has been described previously as a fusion protein with a linking region between the C-terminal region of the b-subunit and the N-terminal region of the δ-subunit with the δ-subunit component bound noncovalently to the N-terminal regions of the three α-subunits (14). However, as described below, this description is inaccurate. In the current structure, the bδ-subunit consists of 16 α-helices with a β-strand separating bδH14 and bδH15, with three additional β-strands between bδH15 and bδH16 (Fig. 5 *A* and *B*). These structural elements form three separate domains. The N-terminal “b” domain is similar to those of other bacterial b-subunits (see *SI Appendix*, Fig. S17). Its structure consists of bδH1 to bδH3 and is similar to the equivalent region of a canonical bacterial b-subunit with bδH1 spanning the bacterial IPM. α-Helix bδH1 and the equivalent, but nonassociated, b′H1 bind to separate regions of the a-subunit and help to maintain the integrity of the transmembrane proton pathway. α-Helix bδH2 extends vertically from the IPM and then runs along the external surface of the F_1_-domain, and finally links to bδH3, which extends the PS to the top of the F_1_-domain. Then, bδH4 to bδH8 form a separate domain, named the “linking domain,” which is attached to bδH9 in the third domain, named “the δ-domain,” consisting of bδH9 to bδH16. The δ-domain is similar to the canonical δ-subunits in other eubacteria (*SI Appendix*, Fig. S18). However, a feature not remarked on previously is that the bδH4 to bδH8 linking domain can be superimposed onto the δ-domain by a clockwise rotation of *ca.*120° (see Fig. 5*C* and Movie S7). Therefore, the δ- and linking domains appear to have arisen by a process that involves a gene duplication event at some point during evolution. The amino acid sequences of the two domains are *ca*. 20% identical, and a further 20% of residues are substituted conservatively (*SI Appendix*, Fig. S19). In the structure, there was no density for residues 1 to 21 of subunit b′ although they are present in the assembled enzyme (*SI Appendix*, Table S1). Thus, the bδ-subunit has two structurally related domains and each binds to the N-terminal region of an α-subunit (subunits α2 and α3) in a similar fashion with the third α-subunit attached by a different mode (Fig. 5 *D*–*F*). In other bacterial species, the mode of binding of the N-terminal region α2 differs from the mode of binding in the *M. smegmatis* ATP synthase, as illustrated in *SI Appendix*, Fig. S20. At present, it is not clear what advantage, if any, is associated with the similarity of the modes of binding of N-terminal regions of α2 and α3.

### Proton Translocation.

**Fig. 6.**
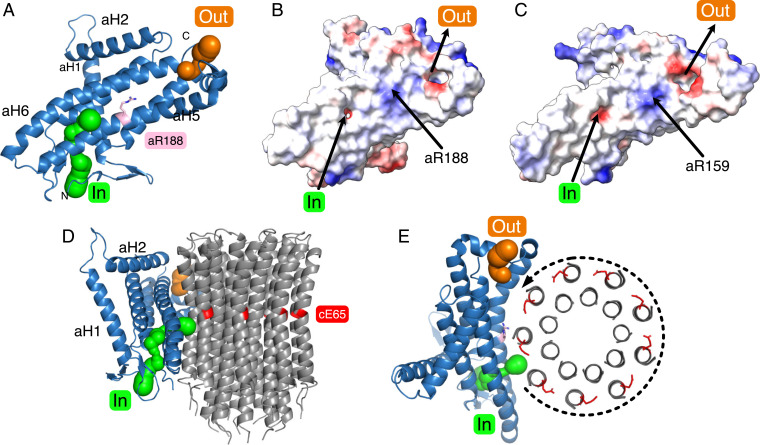
Proton half-channels in the ATP synthase from *M. smegmatis*. (*A*) The a-subunit (blue) viewed from approximately the center of the c_9_-ring toward the lipid bilayer. The inlet and outlet proton half-channels depicted by green and orange spheres, respectively, contain polar residues in the otherwise hydrophobic protein. The essential residue aArg188, located between the two half-channels, deprotonates the carboxylic groups of cGlu65 residues as they are rotated into the inlet half-channel. (*B* and *C*) Comparison of the electrostatic surfaces of the a-subunits from *M. smegmatis* and *Bos taurus*, respectively, illustrating the similar locations of the inlet and outlets of the two half-channels. The electrostatic range is −10 (red), 0 (white), +10 (blue). (*D* and *E*) Views, respectively, of the a-subunit (blue) from inside and from above the plane of the bacterial IPM from the cytoplasmic side showing its spatial relationship to the c_9_-ring (in cartoon representation in *D* and showing cross-sections of α-helices in *E*). Protons enter the inlet channel (green) from the periplasm, passing between α-helices aH5 and aH6 and onto the negatively charged carboxyl of a cE65 residue. The protonation of a cE65 at the inlet is accompanied by the deprotonation of a cE65 residue in the outlet half-channel. The application of the pmf to the c_9_-ring via the Grotthuss water chain in the inlet channel (not resolved) rotates the c_9_-ring in the anticlockwise direction as viewed from above.

One of the most important and fundamental mechanistic features resolved in the recent structure of the dimeric bovine ATP synthase is the Grotthuss chain of water molecules in the inverted L-shaped proton inlet half-channel that leads from the intermembrane space of the mitochondria to the essential γ-carboxylate found on the c-subunit lying at the end of this half-channel (15). This Grotthuss water chain allows the full effect of the transmembrane proton motive force to be applied directly and tangentially to the c-ring to drive its rotation in the direction required for ATP synthesis. In the mycobacterial enzyme, this inverted L-shaped half-channel is clearly present (Fig. 6), but the water molecules that form the Grotthuss chain were not resolved. The mycobacterial inlet channel in the a-subunit contains the polar residues aHis12, aHis15, aHis16, aAsp30, aAsn104, aGln112, aAsp117, aGlu122, aLys125, aGln192, aLys219, aAsp222, and aGln229, which are probably involved in the coordination of the water molecules that form the Grotthuss chain (*SI Appendix*, Fig. S21). The proton inlet pathway then passes through a small aperture between α-helices aH5 and aH6 formed by residues aGly196 and aAsp222 into the interface between the a-subunit and the c-ring. A gap leading to this channel is plugged by residue Gln7 of the b-subunit and another, between aLeu111 and aTyr124, leads to the membrane and is presumably blocked by lipids (*SI Appendix*, Figs. S22 and S23). As in the bovine and other ATP synthases, the outlet channel is more open and funnel-shaped. Polar residues aHis146, aArg153, aLys161, aHis166, aAsn174, aGlu177, aGlu178, aLys181, aSer184, aTyr240, aGln243, and aGlu246 are found in this region. Although the positions and overall polarities of the inlet and outlet half-channels are conserved in mitochondrial and bacterial ATP synthases, in general specific polar residues are not conserved, with the exception of aArg188, and aGly196 found at the aperture between the two arms of L-shaped inlet half-channel plus aAsn192 close to aGly196 and aArg188, and aTyr240 which is between aArg188 and the other residues of the outlet channel (see Fig. 6 and *SI Appendix*, Fig. S21).

### Potential Drug Targets.

The present structure of the ATP synthase from *M. smegmatis* confirms the site of binding of BD described previously and also defines three unique features that have the potential to be exploited in the design of new inhibitors that could be developed into novel drugs against TB. The first of these sites involves the “hook” and “catch” features involved in the ATP hydrolytic inhibitory mechanism, described also in the prior structure. The second is the “fail-safe” device that is likely to enhance the engagement of the hook and loop in state S1. The third is the unique mode of association of the PS with the N-terminal regions of the α-subunits. The “hook and catch” device could be locked in place by small molecules that bind to both features, thereby preventing synthesis of ATP. The “fail-safe” interaction could either be augmented by a small molecule binding across the feature, or its formation could be impeded by small molecules binding to its two elements. Compounds with the features of suitably designed “molecular glues” (29) might enhance the stability of both features and be effective inhibitors of ATP synthesis by the enzyme. The third target would require small molecules that would impede the attachment of the PS to the F_1_-domain during its assembly, thereby preventing the coupling of the pmf to the synthesis of ATP. Once such inhibitors had been identified, then would follow the extensive process of converting them into effective drugs for treating TB.

## Materials and Methods

The mycobacterial ATP synthase was overexpressed in *M. smegmatis* mc^2^4517 from a plasmid containing the entire *atp* operon from *M. smegmatis* mc^2^155 modified to encode a b′-subunit with a C-terminal His_10_-tag. The enzyme was extracted from broken cells with buffer containing 1% (wt/vol) 4-trans(4-transpropylcyclohexyl)-cyclohexyl-α-maltoside and purified by metal affinity chromatography. The subunits of the enzyme were characterized by mass spectrometry. The purified enzyme was applied to electron microscopy grids, and high-resolution cryo-EM data were collected with a Titan Krios instrument. ATP synthase particles were picked with crYOLO (30), and a variety of related structures were determined by hierarchical classification and refinement with RELION (31). The resolution and interpretability of specific regions of the maps were improved by focused local refinement of the membrane domain, the membrane extrinsic catalytic domain, the PS, the stator and the bδ-subunit, and these subregion reconstructions were assembled together into composite structures and refined into atomic models. Merging of substate particle sets at the expense of substate distinction improved the resolutions in the F_1_, membrane, rotor, and bδ domains, and reclassification of all particle data at the expense of rotational state distinction, while masking the membrane domain, revealed the presence of BD. Fourier shell correlation curves and local resolution estimations were calculated with RELION. Model building into focused maps was performed with Coot (32), and real space refinement with PHENIX (33–35). The starting model comprised the crystal structures of the F_1_-domain of *M. smegmatis* ATP synthase (PDB ID code 6FOC) (6) and the c_9_-ring in the presence (PDB ID code 4V1F) and absence of BD (PDB ID code 4V1G) (5). All other subunits were built de novo. Model geometry and density fit validation were performed by MolProbity (36, 37) and EMRinger (38), respectively.

## Supplementary Material

Supplementary File

Supplementary File

Supplementary File

Supplementary File

Supplementary File

Supplementary File

Supplementary File

Supplementary File

## Data Availability

Protein models and electron density map data have been deposited in the Protein Data Bank and the Electron Microscopy Data Bank under the following accession numbers: 7NJK (EMD-12377–12381), 7NJL (EMD-12382–12386), 7NJM (EMD-12387–12391), 7NJN (EMD-12392–12396), 7NJO (EMD-12397–12401), 7NJP (EMD-12402–12406), 7NJQ (EMD-12407–12411), 7NJR (EMD-12412–12416), 7NJS (EMD-12417–12421), 7NJT (EMD-12422), 7NJU (EMD-12423), 7NJV (EMD-12424), 7NJW (EMD-12425), 7NJX (EMD-12426), 7NJY (EMD-12427), 7NK7 (EMD-12432), 7NK9 (EMD-12434), 7NKB (EMD-12436), 7NKD (EMD-12438), 7NKH (EMD-12439), 7NKP (EMD-12404), 7NKK (EMD-12442), 7NKL (EMD-12406), 7NKJ (EMD-12441), 7NL9 (EMD-12461), 7NKN (EMD-12444), and 7NKQ (EMD-12446).
