# Supplementary material for: Structure of the ATP synthase from Mycobacterium smegmatis provides targets for treating tuberculosis
Source: Proc Natl Acad Sci U S A. 2021 Nov 15;118(47):e2111899118. doi: 10.1073/pnas.2111899118 (PMC8617483; doi:10.1073/pnas.2111899118)
Supplement: Supplementary File [file pnas.2111899118.sapp.pdf]

PNAS
www.pnas.org

Supplementary Information for

Structure of the ATP synthase from *Mycobacterium smegmatis* provides targets for treating tuberculosis

Martin G. Montgomery[a1], Jessica Petri[a1], Tobias E. Spikes[a1], and John E. Walker[a,2]
[a]*The Medical Research Council Mitochondrial Biology Unit, University of Cambridge, Cambridge Biomedical Campus, Hills Road, Cambridge CB2 0XY, United Kingdom*

[1] Equal contribution
[2]Corresponding author: John E. Walker
Email: walker@mrc-mbu.cam.ac.uk

This PDF file includes:

Supplementary text
Figures S1 to S23
Tables S1 to S4
Legends for Movies S1 to S7
SI References

Other supplementary materials for this manuscript include the following:

Movies S1 to S7

Supplementary Materials and Methods.

Biochemical Methods

Protein concentrations were measured by the Pierce™ BCA Protein Assay Kit (Thermo Fisher Scientific). ATP synthase was analyzed by SDS-PAGE on 12-22% Tris-glycine gels. Protein bands were detected by staining with Coomassie-Blue dye and were excised from the gel. The identities of the proteins were verified by mass mapping of tryptic and chymotrypsin peptides by matrix assisted laser desorption ionization-time of flight mass spectrometry.

Protein Expression and Purification

Expression plasmid pYUB-E2 (derived from pYUB28b (1)) containing a T7 promotor, Lac operator and T7 terminator, had the full *atp* operon from *M. smegmatis* mc^2155 with a His_{10}-tag at the C-terminus of subunit b' inserted and was transformed into *M. smegmatis* mc^24517. The cells were grown at 37°C to an optical density of 0.5-1.0 at 600 nm in 2xTY medium plus 0.05% (w/v) Tween 80, hygromycin B (50 $\mu g ml^{-1}$) and kanamycin (20 $\mu g ml^{-1}$). Expression from the T7 promoter was induced with 1 mM isopropyl-β-D-1-thiogalactopyranoside and the culture was incubated for 24 h at 28°C. Then the cells were harvested by centrifugation (5,310 x g, 25 min, 4°C), washed with buffer (50 mM Tris-HCl pH 7.5, 5 mM $MgCl_2$ and 0.05% [w/v] Tween 80) and either used immediately or stored at -20°C. Cells (ca. 80-90 g) were resuspended in the same buffer plus cOmplete™ EDTA-free protease inhibitor tablets (Roche) and DNase I (Roche), and dispersed by sonication (5 cycles; amplitude 30; 20 secs with intervening 1 min pauses). Then the cells were disrupted by three passages through a Constant System cell disrupter at 20 kpsi. Cell debris was removed by centrifugation (10,000 x g for 20 min at 4°C), and the supernatant was centrifuged again (131,500 x g for 45 min at 4°C). The pellet was resuspended in the same buffer to a protein concentration of 5 mg/ml and extracted with 1% [w/v] trans-PCC-α-maltoside (t-PCCαM; Glycon Biochemicals GMbH) for 1.5 h at room temperature. Insoluble material was removed by centrifugation (131,500 x g for 45 min at 4°C). The supernatant was adjusted by addition of NaCl and imidazole to 150, and 20 mM, respectively, and then loaded onto a HisTrap HP column (5 ml; GE Healthcare) equilibrated in buffer A (50 mM Tris-HCl pH 8.0, 150 mM NaCl, 20% [w/v] glycerol, 5 mM $MgCl_2$, 1 mM tris(2-carboxyethyl)-phosphine, 1 mM ADP, 20 mM imidazole, 0.1% [w/v] t-PCCαM and 0.1 mM phenylmethylsulfonyl fluoride) at a flow rate of 1.5 ml/min. The ATP synthase was eluted with a linear gradient of buffer A (100 ml) and buffer B (buffer A containing 500 mM imidazole; 100 ml) at a flow rate of 1.0 ml/min. Fractions containing the enzyme were pooled and dialysed for 18 h at 4°C in buffer containing 50 mM Tris-HCl, pH 8.0, 150 mM NaCl, 20% [w/v] glycerol, 2 mM $MgCl_2$, 1 mM tris(2-carboxyethyl)-phosphine, 1 mM ADP, 0.1% [w/v] t-PCCαM and 0.1 mM phenylmethylsulfonyl fluoride. The sample was loaded onto a HisTrap HP column (1 ml; GE Healthcare) equilibrated in buffer A. ATP synthase was eluted with buffer A containing 250 mM imidazole. The sample of enzyme was concentrated by centrifugal ultrafiltration (100 kDa molecular mass cut-off) and then loaded onto a Superdex 200 increase 5/150 GL column (GE Healthcare) equilibrated with size exclusion buffer (50 mM Tris-HCl pH 8.0, 150 mM NaCl, 2 mM $MgCl_2$, 1 mM tris(2-carboxyethyl)-phosphine, 1 mM ADP, 0.1% [w/v] t-PCCαM and 0.1 mM phenylmethylsulfonyl fluoride) at a flow rate of 0.1 ml/min.

Mass Spectrometric Analysis of Intact Subunits of ATP synthase from *M. smegmatis*

The purified enzyme (ca. 45 μg) was precipitated with ethanol and redissolved in a solution containing 60% (v/v) formic acid, 15% (v/v) trifluoroethanol, 1% (v/v) hexafluoro-isopropanol and 1 mM tris-(2-carboxyethyl)-phosphine (TCEP). The subunits were introduced onto a PLRP-S column (Agilent; 1.0 x 75 mm; particle size 1000 Å) equilibrated in a buffer containing 50 mM ammonium formate, pH 3.1, 10% (v/v) trifluoroethanol and 1% (v/v) hexafluoroisopropanol at flow rate of 50 μl/min, and eluted with a gradient of 2-propanol. The effluent was introduced directly into the electrospray interface of a SciEx Q-Trap 4000 triple quadrupole mass spectrometer operated in positive ion mode (Table S1).

Preparation of Grids for Cryo-EM.

The enzyme was concentrated by ultrafiltration (100 kDa molecular mass cut-off) to 4.2 mg/ml. Bedaquiline (solubilized in 0.5% DMSO) was added to a final concentration of 25 μM. Both sides of UltrAUFoil (R1.2/1.3 mesh, Quantifoil Micro Tools GmBH) TEM supports were glow discharged at 25 mA under vacuum for 30 sec. The supports were then put into tubes (0.5 ml) containing a small portion of an ethanolic solution of 5 mM mercaptopoly(ethyleneglycol)carboxylic acid (PEG-thiol) (2). The tubes were flushed with gaseous N_2, sealed and stored in a secondary N_2 flushed

container for at least 48 h. Then the grids were washed three times with absolute ethanol and dried in air. 3 µl sample was applied to the pegylated UltrAUFoil gold grids at room temperature and 100% relative humidity, left to distribute for 15 s, and blotted for 8.5 s at a force setting of -10 with an FEI Vitrobot Mark IV instrument. The grids were then vitrified in liquid ethane.

Cryo-EM Data Collection

A high-resolution dataset was collected with a Titan Krios electron microscope operated at an accelerating voltage of 300 kV and equipped with a K3 direct electron detector (Gatan) and post-column imaging energy filter set to a slit width of 20 eV. Images were collected in super-resolution electron counting mode using the SerialEM software package for automated data collection. A total of 11,225 movies were recorded at a nominal sampling rate of 0.83 Å/pixel. Dose-fractioned 2.5 s movies of 41 frames were recorded with total and per frame doses of *ca.* 60 $e^-/Å^2$ and 1.46 $e^-/Å^2$, respectively. The instrument was located at the eBIC (electron bio-imaging centre) at the Diamond Light Source, Harwell Campus, Oxford, U.K.

Cryo-EM Image Processing

Image analysis was performed with RELION-3.1 (3), unless otherwise specified. First, the gain reference was applied to the raw movie frames and beam-induced motion and stage drift were corrected with MotionCorr2 without dose weighting. Initial CTF parameters were determined from the motion corrected frame sums with CTFFIND4.1 (4). Images displaying significant drift, or where Thon rings were not fitted reliably below 6.0 Å, were removed by visual inspection and curation, as were others with non-vitreous ice or significant contamination. Automated particle picking was carried out with the neural network-based particle picker crYOLO. The picking model was pre-trained with *ca.* 1,000 manually curated coordinates and identified 437,452 additional coordinates which were extracted at a down-sampled pixel size of sampling rate x 4 and subjected to several rounds of reference free 2D-classification and subset selection (Fig. S2). This procedure yielded 285,681 particles which were re-extracted subsequently at a nominal sampling rate of 0.83 Å/pixel in 500-pixel boxes and refined against a map of the *M. smegmatis* ATP synthase prepared from an independent dataset to produce a consensus reconstruction. Then, these particles were classified without particle re-alignment using the consensus orientations determined by the previous refinement in order to separate particles by rotational state as defined by the asymmetry of the central stalk. This procedure yielded two classes representing 81,676 and 46,667 particles in rotational state 1, one class representing 24,938 particles in rotational state 2, one class representing 85,548 particles in rotational state 3 and a 5th class, comprised of 46,852 images, that were discarded because the quality of the resulting reconstruction was poor. Further classification of the discarded particles did not yield any sub-sets of defined rotational state.

For each of the four retained classes, per-particle CTF parameters were determined with RELION-3.1 (3), and particles, with their associated per-particle defocus estimates, were subjected independently to Bayesian polishing and subsequent iterations of refinement and per-particle defocus estimation until no further improvements to the estimated resolutions of the reconstructions were observed. The final iteration included beam-tilt estimation and corrections of higher-order aberrations (3). Magnification anisotropy was not corrected. Bayesian polishing was then repeated before further downstream processing. Refinement of the polished particles yielded 2 reconstructions of the complex in state 1 with estimated resolutions of 2.4 Å and 2.7 Å, and single reconstructions of the complex in states 2 and 3 with estimated resolutions of 3.1 Å and 2.4 Å, respectively. It was apparent from inspection of these reconstructions that significant heterogeneity was still present, resulting in poor density and obvious averaging of features in the membrane domain. Therefore, each of the particle sets was subjected to further stages of classification without particle re-alignment alignment, as above. The number of classes and number of stages required varied within the sub-sets and is summarised in Fig. S3. This hierarchical classification procedure yielded a total of 9 ostensibly homogenous sub-states across the 3 major rotational states with estimated resolutions of 2.52-4.15 Å. In some cases, sub-classes were discarded due to low particle numbers, poor reconstruction quality or both (see Fig. S3, denoted 'X').

Then the quality of the maps and the resolution of specific regions were improved for each sub-state by focussed local refinement of the catalytic domain, the membrane domain, the stator and the b-delta fusion domain (see Fig. S3). Masks were created for the catalytic domain (the $\alpha_3\beta_3$ hexamer plus the rotor domain subunits γ and ε); the membrane domain (subunit a, the transmembrane portions of subunits bδ and b' and the c_9-ring); the stator (the PS subunits b', bδ

plus portions of the membrane associated a-subunit); and the bδ-subunit, and the masks were employed during refinements, initialised from the previously determined consensus orientations for each sub-state, with restricted angular and translational searches of 0.5^{o} and 3 pixels, respectively. The reconstructions are summarised in Fig. S3 and Table S3. Many of these were used during the initial model building process and are deposited as described by Table S3. Preliminary composite maps of each sub-state were assembled by rigid body fitting of the reconstructions of each local region into its respective consensus reconstruction in UCSF ChimeraX (5) and subsequent application of the 'volume maximum' command. Initial atomic models were built into these composites with a focus on Cα-trace accuracy and the composites recreated using the initial Cα model to select the regions of interest with the 'volume zone' and 'volume maximum' commands in ChimeraX. These more accurate composite maps are shown in Fig. S3, deposited as described in Table S3 and used for atomic model refinement of the complete/intact substates.

A similar focussed local refinement procedure was applied also to combinations of sub-states grouped by rotational state. This procedure improved the resolutions of these regions at the expense of substate distinction. In these cases, masks were supplied of the $\alpha_3\beta_3$ hexamer, the membrane domain (as above), the intact rotor (subunits γ, ε and the c_9-ring) and the bδ-subunit (as above). Similarly, a combined set of all particles was employed to analyze the membrane domain at the expense of rotational state distinction. The membrane domains of all particles were refined whilst employing the membrane domain mask and restricted angular searches, before classification without particle re-alignment into five classes (see Fig.S3 and Table S3.). Where resolutions are reported, half-maps were masked with soft-edge masks with cosine edges and the resolutions of the reconstructions were estimated in RELION (3). The effect of the mask was deconvoluted by a phase-randomisation procedure to provide an unbiased estimate and *ad hoc* B-factors were applied according to the appearance of the density in the reconstructions, unless otherwise stated. We observed that the automated procedure for the estimation of map B-factors (6) tended to over sharpen the reconstructions in this dataset.

Modelling and refinement of the structure of ATP synthase from *M. smegmatis*

Model building into focussed maps was performed with COOT (7) and real space refinement with PHENIX (8). The starting models comprised the following crystal structures of sub-domains of *M. smegmatis* ATP synthase; F_1-ATPase (PDB6FOC) (9), and the c_9-ring in the presence (PDB4V1F), and absence of bedaquiline (PDB4V1G) (10). All other subunits were built *de novo*. Various cycles of real space refinement and model building were performed with Phenix (11), and COOTt (7), respectively. Stereochemistry of the structures were assessed with MolProbity (12) and the model to map fit by PHENIX (8) and EMRinger (13).

Structural Analysis

Channels in the a-subunit were analyzed using MOLE (14). Sequence alignments were performed by clustal O via www.uniprot.org.

Figures, movies and animations

Figures and movies were prepared with USCF ChimeraX (5) and PyMOL (15).

Supplementary Results

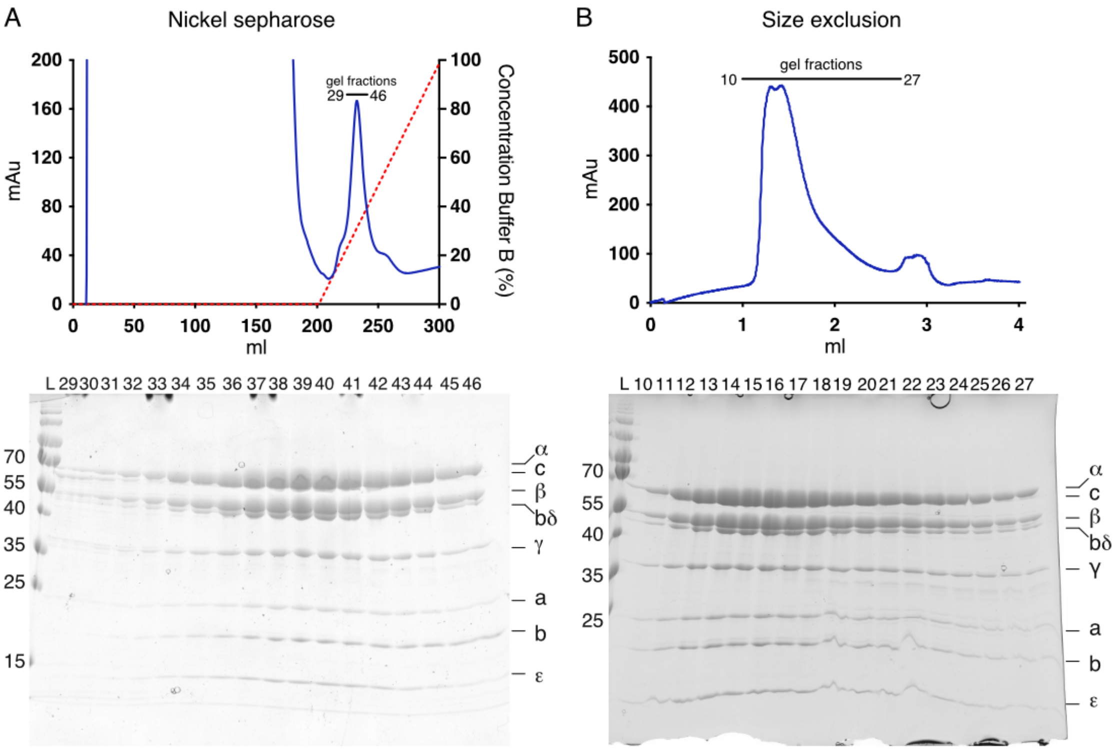

Fig. S1. Purification of the ATP synthase from *M. smegmatis*. The enzyme was purified by nickel affinity chromatography via a His_{10} tag at the C-terminus of the b-subunit and by size exclusion chromatography. *A,* Nickel Sepharose chromatography. The absorbance trace is blue and the gradient of increasing imidazole concentration is indicated by the red dotted line. *B*. Size exclusion chromatography on a Superdex 200 increase 5/150 GL column. Beneath *A* and *B* are shown the SDS-PAGE analyses of the fractions indicated by the horizontal bars. Molecular weight markers (kDa) are shown on the left of each gel and the identities of the subunits are indicated on the right.

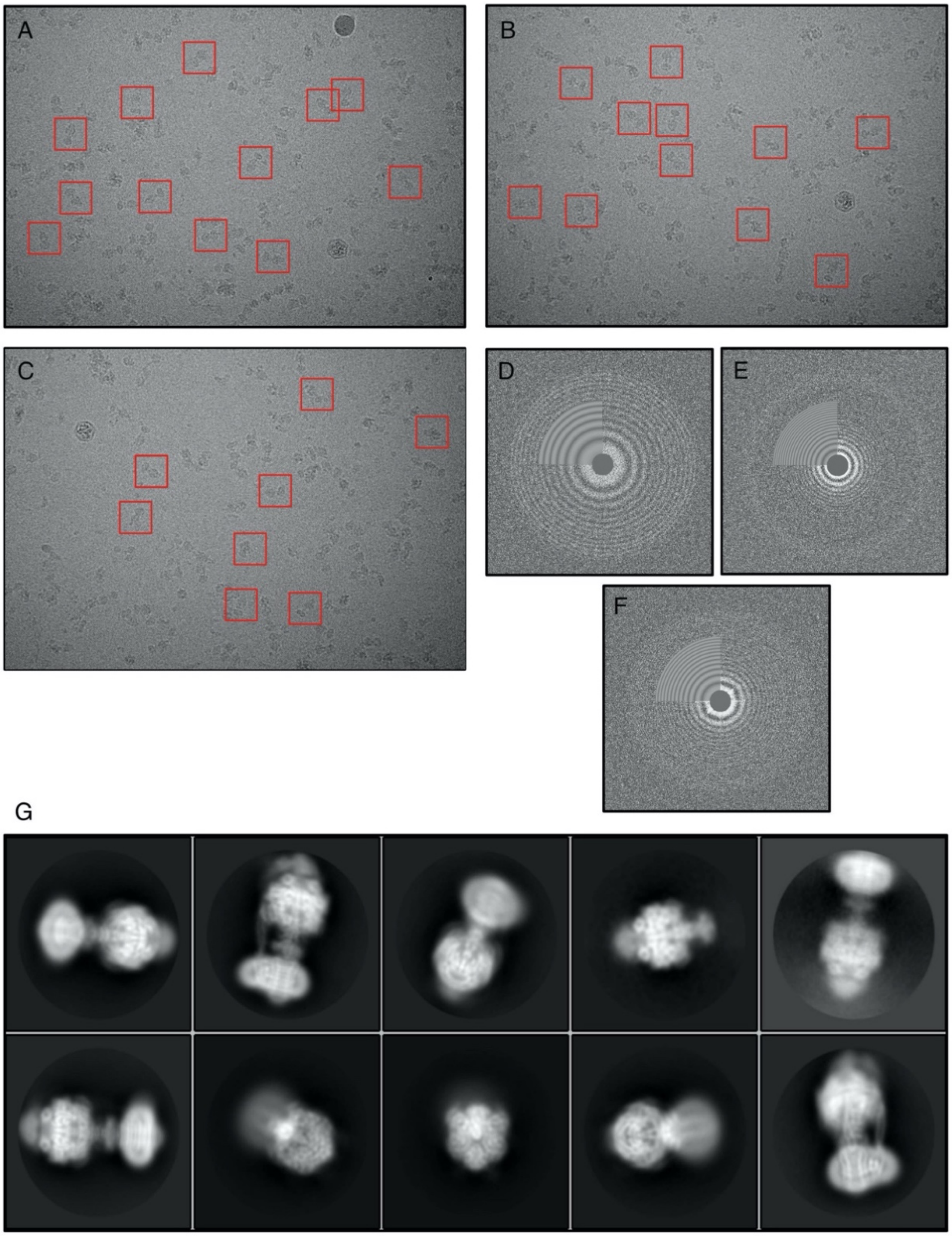

Fig. S2. Representative cryo-em images of the ATP synthase from *M. smegmatis*. *A-C,* purified samples of the ATP synthase with examples of particles in red boxes. *D-F,* representative radial power spectra of micrographs collected in *D* at low defocus (800 nm), in *E* at high defocus

(2200 nm), and in *F* at intermediate defocus (1550 nm), with Thon rings visible to at least 3 Å. The fit quadrant shown extends to 5 Å. In most cases, Thon rings were fitted accurately at a resolution of *ca.* 2.6 Å by CTFFIND-4.1 (4). *G,* representative 2D-class averages in various orientations in the vitreous ice layer calculated at a pixel size of 3.32 Å/pix.

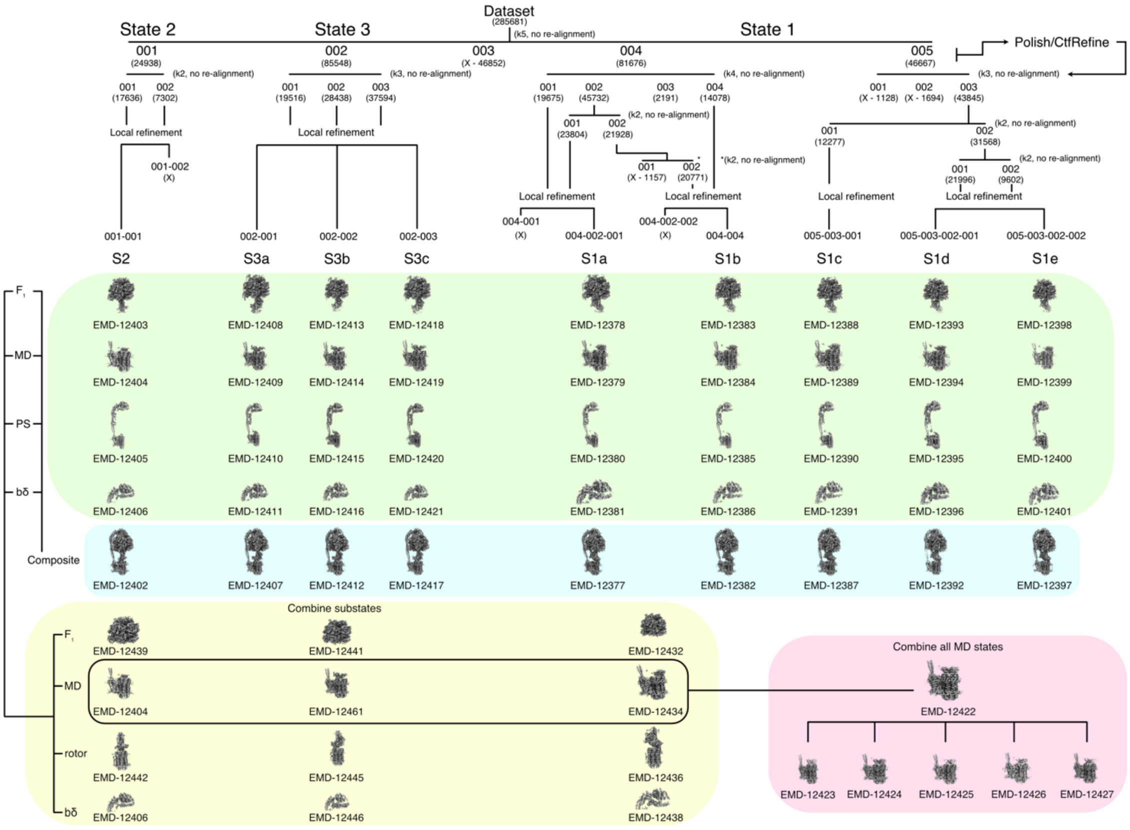

Fig. S3. Particle classification and map generation. Particles (numbers in parentheses) were divided into five main classes (001-005) which were assigned to catalytic states as follows: State1, classes 004 and 005; State 2, class 001; State 3, class 002. Class 003 had too few particles to yield useful data. The main classes were subdivided into a total of nine substates, named s1a-e, s2, and s3a-c. Domain focussed local refinement of the particles yielded maps (green section) that were combined into composite ATP synthase maps for each substate (blue section). Within each substate, the green section particle sets were combined to improve the resolution of the subdomains at the cost of substate distinction (yellow section). By performing Membrane Domain focussed refinement on all particles, maps (red section) were obtained that allowed BD to be built.

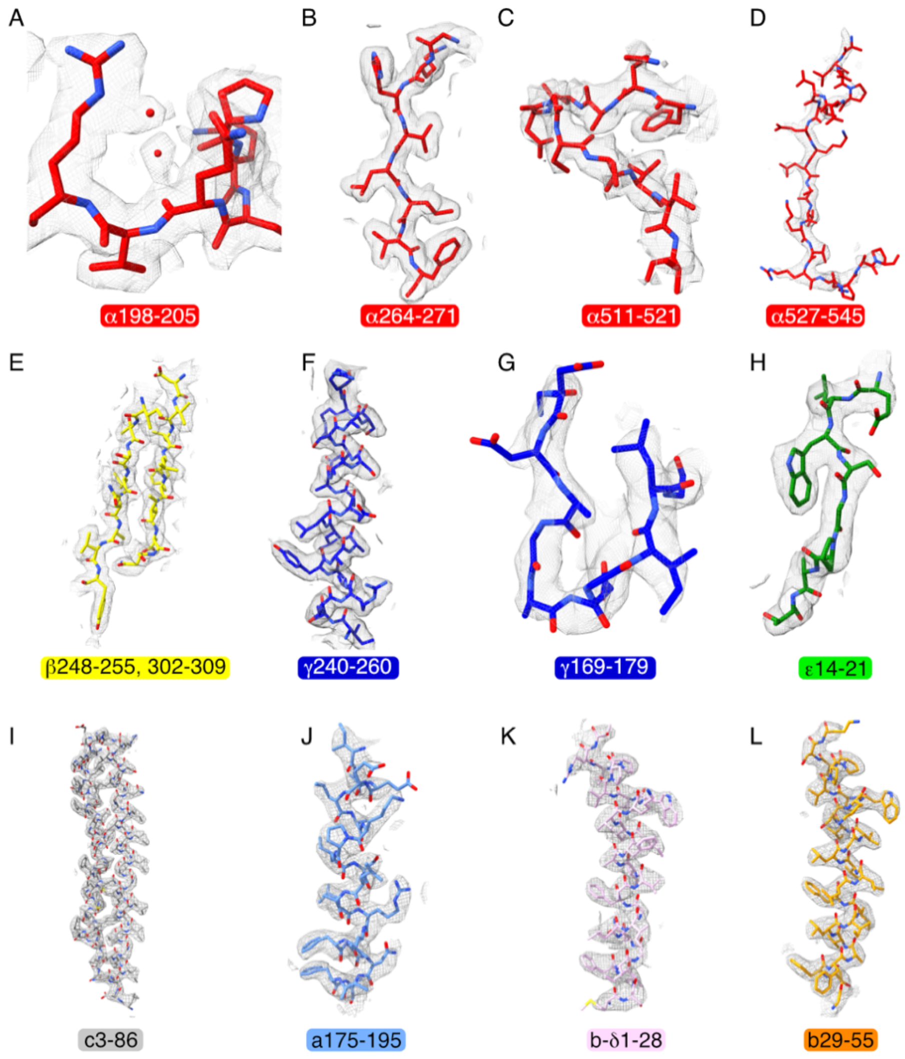

Fig. S4. Quality of the electron density of subunits of the ATP synthase from *M. smegmatis.* The quality of the density ranges from resolved waters to barely resolved side-chains. *A-C*, F_1 state 1 (EMD-12432, PDB 7NK7). *D*, Rotor state 3 (EMD-12445, PDB 7NKO). *E*, F_1 state 1 (EMD-12432, PDB 7NK7). *F-H*, Rotor state 3 (EMD-12445, PDB 7NKO). *I-L*, F_o combined all (EMD-12422, PDB 7NJT).

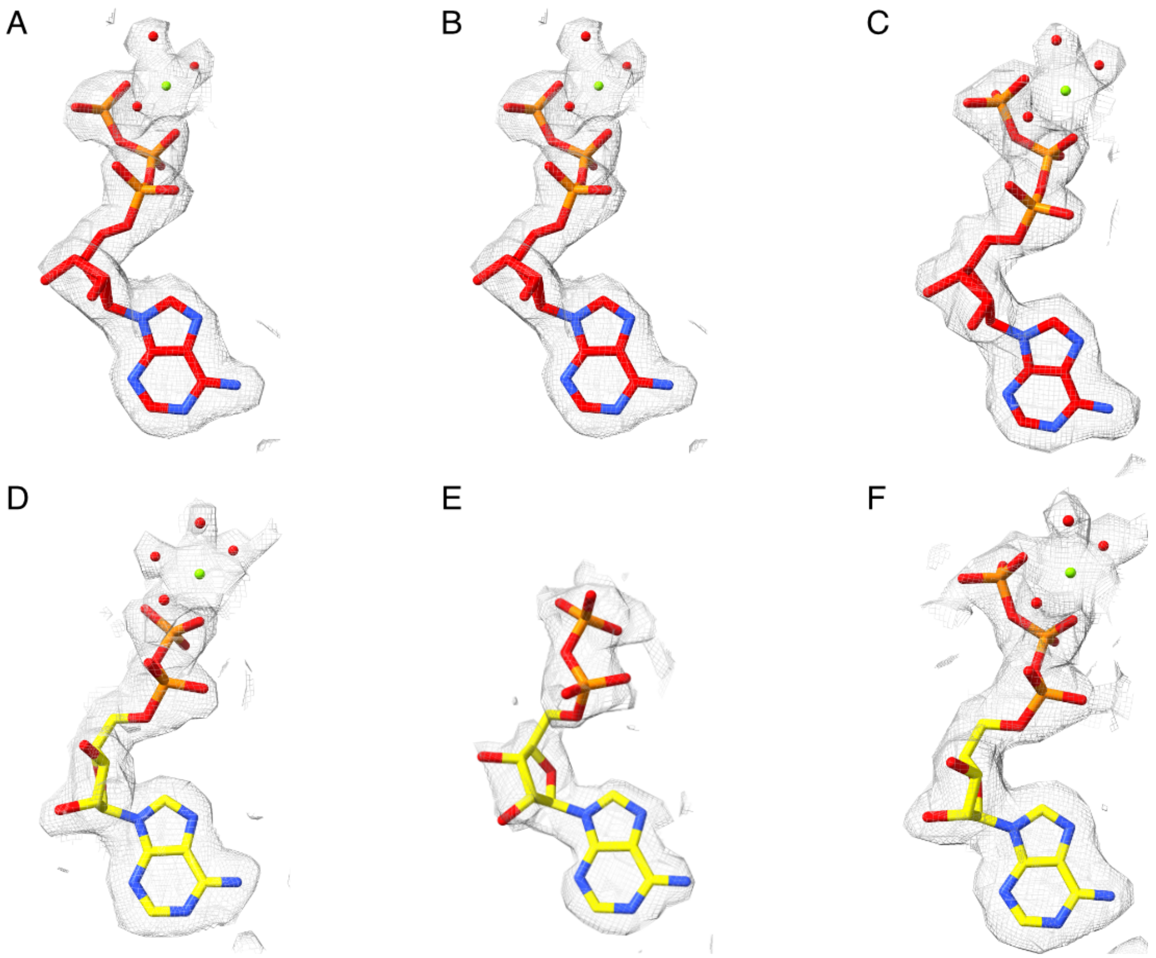

Fig. S5. Electron density for the nucleotides associated with the ATP synthase from *M. smegmatis*. A, α_E (chain A); B, α_{TP}, (chain B); C, α_{DP} (chain C), D, β_{DP} (chain D); E, β_E (chain E); and F, β_{TP} (chain F). F_1 state 1 (**EMD-12432, PDB ID 7NK7)**, map level 0.018V. The Mg^{2+} ions and co-ordinating waters are shown as green and red spheres, respectively.

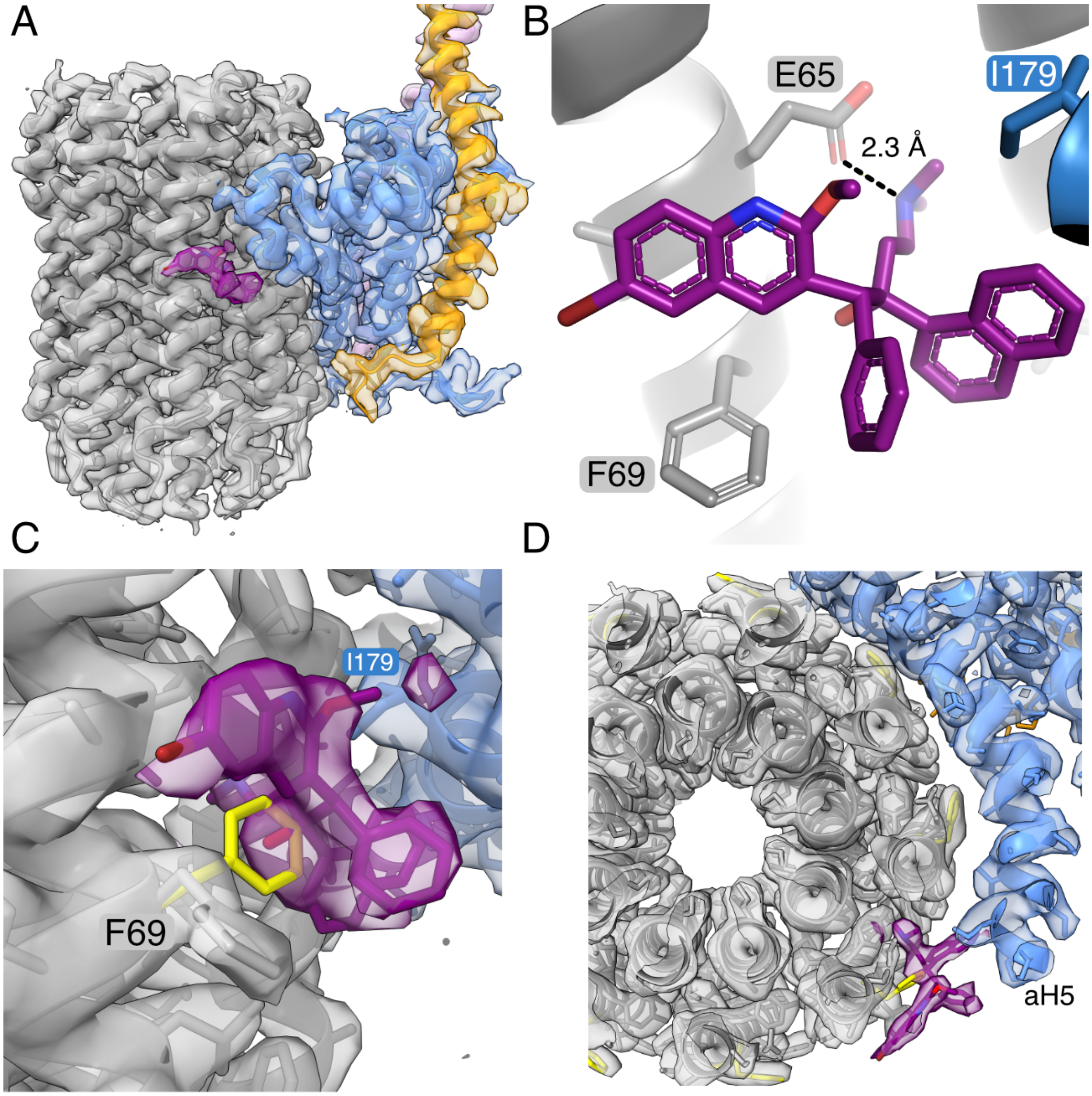

Fig. S6. Binding of bedaquiliine to ATP synthase from *M. smegmatis*. BD was present in a minority of particles and so the corresponding density varies between states and substates. Focused refinement on the membrane domain (MD) of all particles yielded the "MD combined" series of maps (SI Fig 3 and SI Tables 3 and 4) The strongest density was observed in MD combined class 2 (PDB 7NJV, EMD-12424). BD is bound to the c-subunit near to, but not in contact with, the N-terminal region of α-helix aH5, in a position that should inhibit synthesis of ATP. In *A*, the electron density of the membrane domain is shown as a transparent surface with the model shown in cartoon and stick formats. The a-, bδ-, b'-, and c-subunits are sky blue, pink, orange, and grey, respectively. BD is purple. *B*, BD is bound such that its N04 moiety interacts with cGlu65 (oxygen and nitrogen atoms of cGlu65 and BD are red and blue, respectively.), but, as shown in *C* and *D*, its binding requires cPhe69 (yellow) to move from a position in the active enzyme where it is folded against the c-ring to allow the ring to rotate past the a-subunit, and to adopt a non-native conformation where it has rotated downwards by 120° (grey). In D, the c-ring is viewed from above and along the axis of rotation, with synthesis in the anticlockwise direction. BD is shown bound in a position close to the N-terminal region of aH5 that should inhibit synthesis. A BD molecule bound to a c-subunit close to the C-terminal region of aH5 should prevent hydrolysis (not illustrated). Therefore, presence of bound BD should prevent rotation of the c_9-ring in either direction as the a-c interface cannot accommodate its extra bulk.

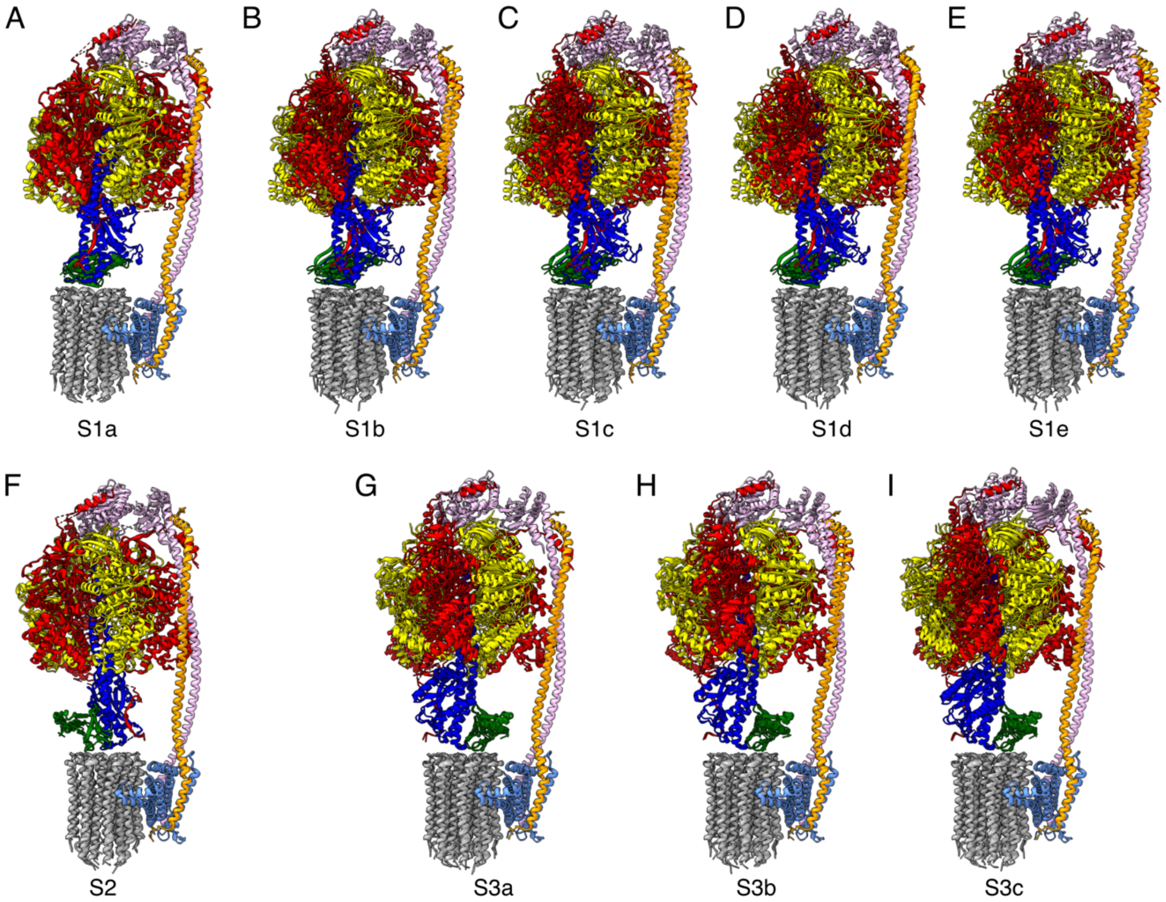

Fig. S7. Structures of *M. smegmatis* ATP synthase. Recent structures depicting the three main states of the ATP synthase from *M. smegmatis* (16) have been aligned via the a-subunit to the nine State/substate structures reported here. Parts *A*-*E*, State 1 (PDB 7JG5) aligned to substate s1a (PDB 7NJK), substate s1b (PDB 7NKL), substate s1c (PDB 7NKM), substate s1d (PDB 7NKN), substate s1e (PDB 7NKO) respectively. S1a is the closest match to previously published main state S1 structure whilst the other substates others show subtle movement in F_1, the c-ring and PS yielding further information on the interco-operativity of subunits during catalysis. Part *F*, State 2 (PDB 7JG6) closely matches s2 (PDB 7NJP). Parts G-I, State 3 (PDB 7JG7) closely matches the F_o domains of s3a (PDB 7NJQ), s3b (PDB 7NJR) and s3c (PDB 7NJS) but there are differences in the F_1 and PS domains. The α-, β-, γ-, ε-, a-, bδ-, b'-, and c-subunits are colored red, yellow, blue, green, sky blue, pink, orange, and grey, respectively.

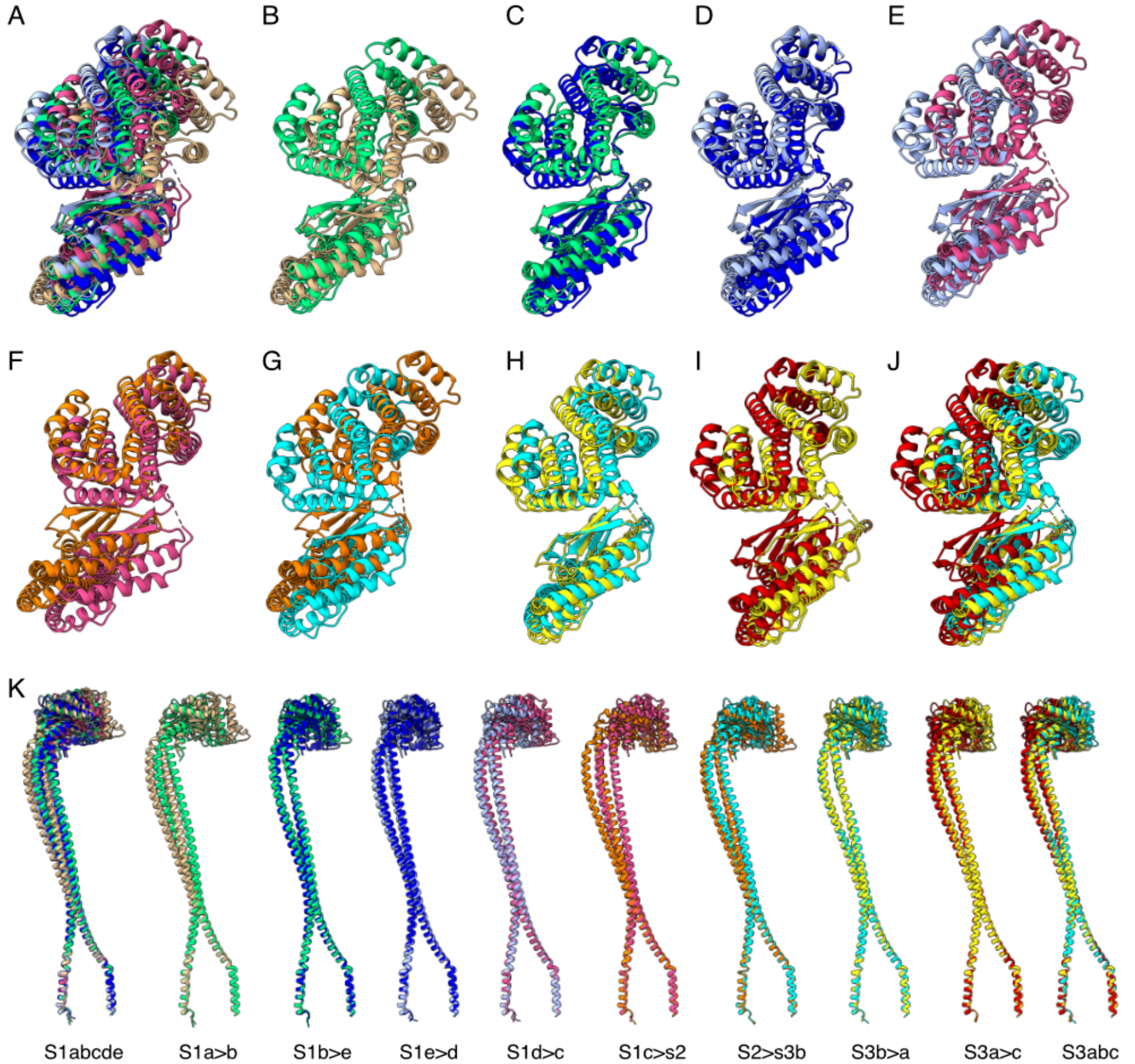

Fig. S8. **Positions of the PS subunits in the substates of States 1 and 3.** Views in A-J, from above, and in *K* from the side of models of the PS of all substates aligned via the a-subunit. s1a-e, are colored tan, green, magenta, pale blue and dark blue, respectively; S2 is orange; s3a-c are yellow, cyan and red, respectively. In *A*, all State 1 substates are shown. In *B-I*, pairs of substates showing the least movement required to arrive at the next substate. *J*, all substates of State 3. *K*, Similar transitions viewed from the side.

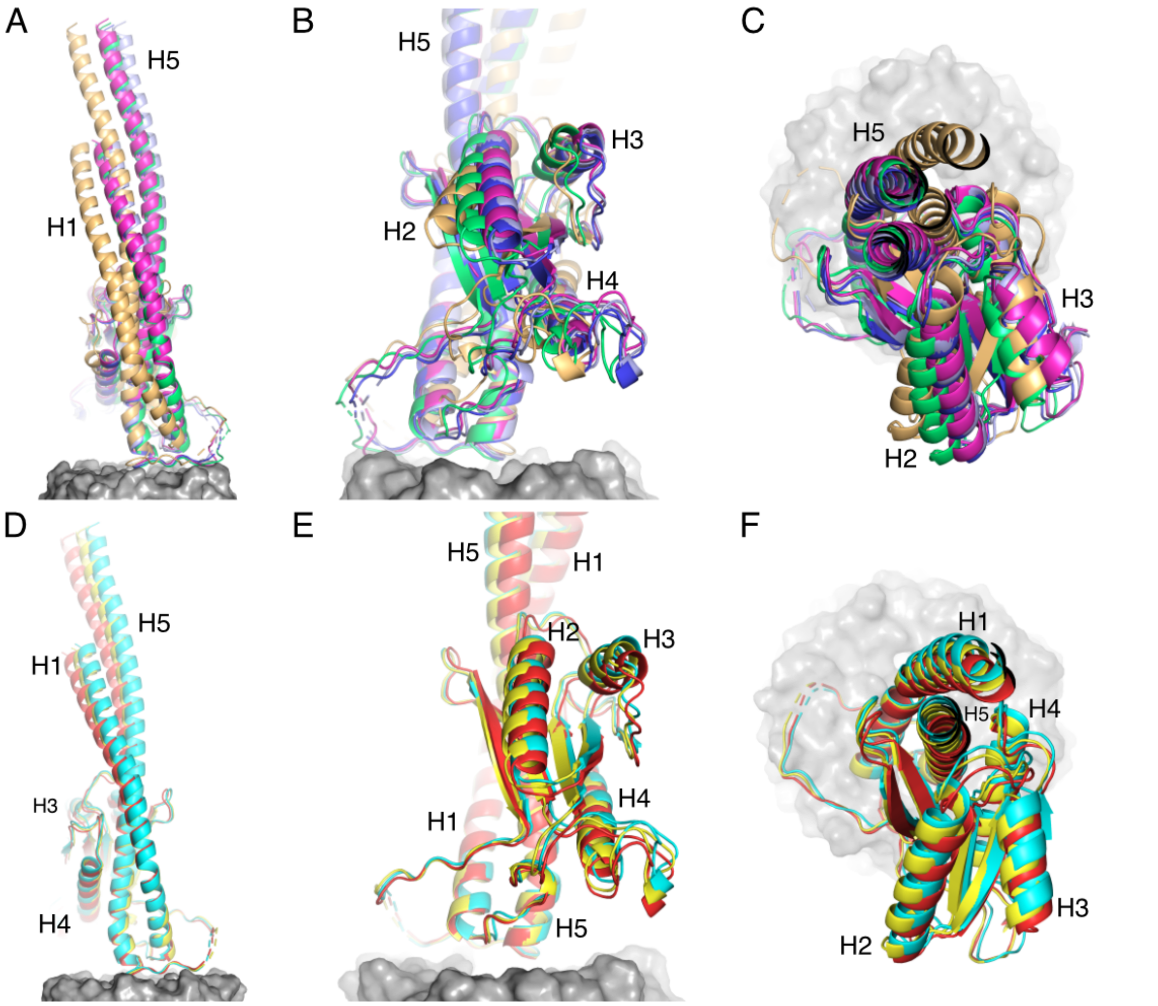

Fig. S9. Positions of the γ-subunit in the State 1 and State 3 substates of the ATP synthase from *M. smegmatis*. The models of State 1 substates s1a-s1e are colored tan, green, magenta, pale blue and dark blue, respectively. The models of state 3 substates s3a-c are colored yellow, cyan and red, respectively. Models are aligned via the a-subunit. Parts *A-C*, state 1 substates, viewed from *A*, in front and *B*, behind the coiled coil, and *C*, a view from above showing the lower half of the γ-subunit. Substate s1a shows a significant displacement compared to the others due to the rotor position being one c-subunit different to s1b-e. Substates s1c-e have the c-rings in similar positions yet there are differences in the γ-subunit increasing from the point that it enters the $\alpha_3\beta_3$ domain of F_1. Parts *D-F*, state 3 substates. Similar to substates s1c-e, despite the c-rings being in approximately the same position, differences in the γ-subunit increase the further the subunit is encased by the $\alpha_3\beta_3$ domain of F_1.

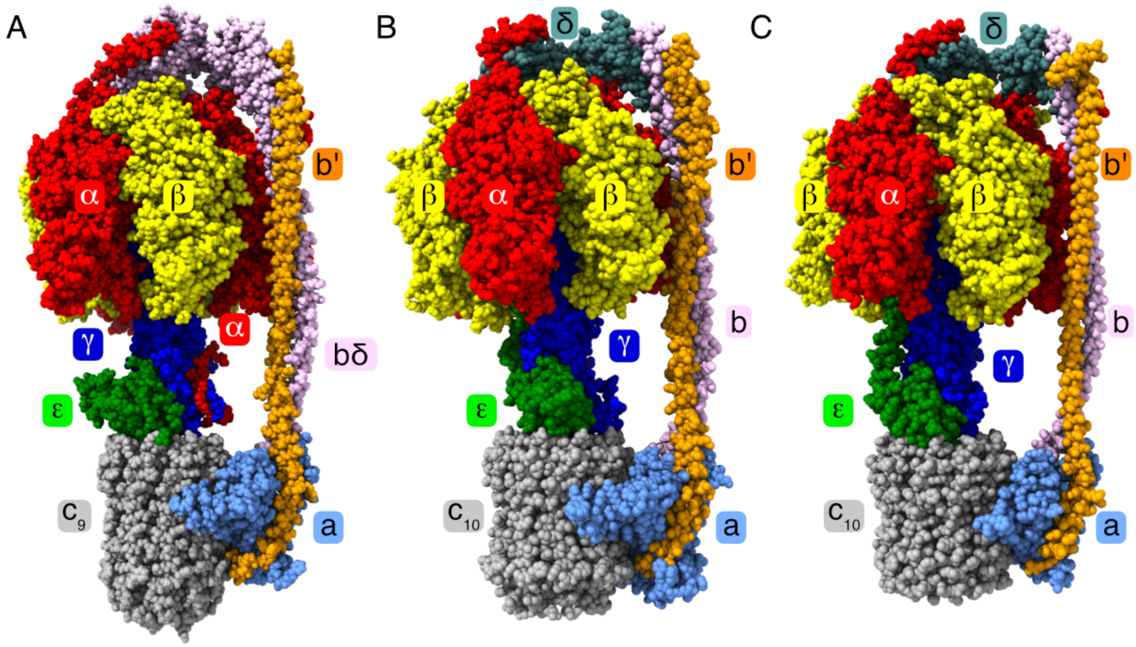

Fig. S10. Comparison of the structures of the ATP synthases from *M. smegmatis*, *E. coli* and *G. stearothermophilus*. A, *M.smegmatis* (PDB 7NJP). B, *E. coli* (PDB 6OQR). C, *G. stearothermophilus* (PDB 6N2Z). Overall, the structures of the enzymes are similar. The main differences between *M. smegmatis* and the two others are as follows: there is one fewer c-subunit in the ring; the C-terminal α-helices of the ε-subunit do not extend into the F_1 domain (which has the effect of twisting the F_1 slightly in *E. coli* and *G. stearothermophilus*); the bδ-subunit is found only in the *M. smegmatis enzyme*.

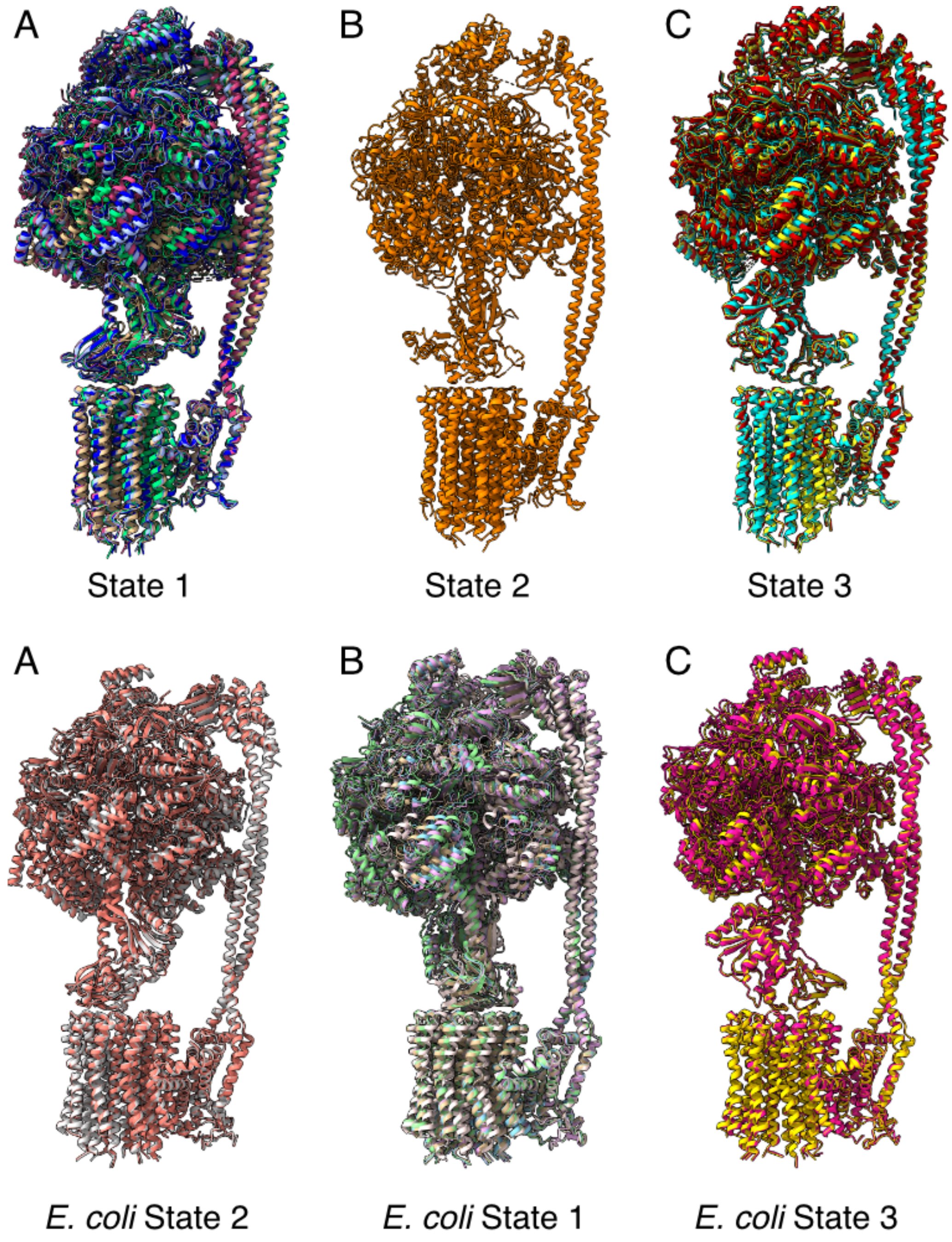

Fig. S10. Comparison of the substates in the catalytic cycles of the ATP synthases from *M. smegmatis* and *E. coli*. Substates have been resolved in both ATP synthases. In both cases, one state has more substates than the others. These states have been designated as State 1, and they correspond to the states with the most particles in the respective datasets. However, the States 1 are different. In *M. smegmatis*, State 1 contains five substates, s1a-e. *E. coli* State 1 also has five substates, s1a-e, but it is the equivalent of State 2 in *M. smegmatis*. Models are aligned via their a-subunits. The lower regions of the enzyme align well in each state, but significant differences are found in the F_1 domain and at the top of the PS. *E. coli* models: State 1 PDB 6OQR-6OQU, 6PQV; State 2 PDB 6WNQ, 6OQV, and State 3 PDB 6OQW, 6WNR.

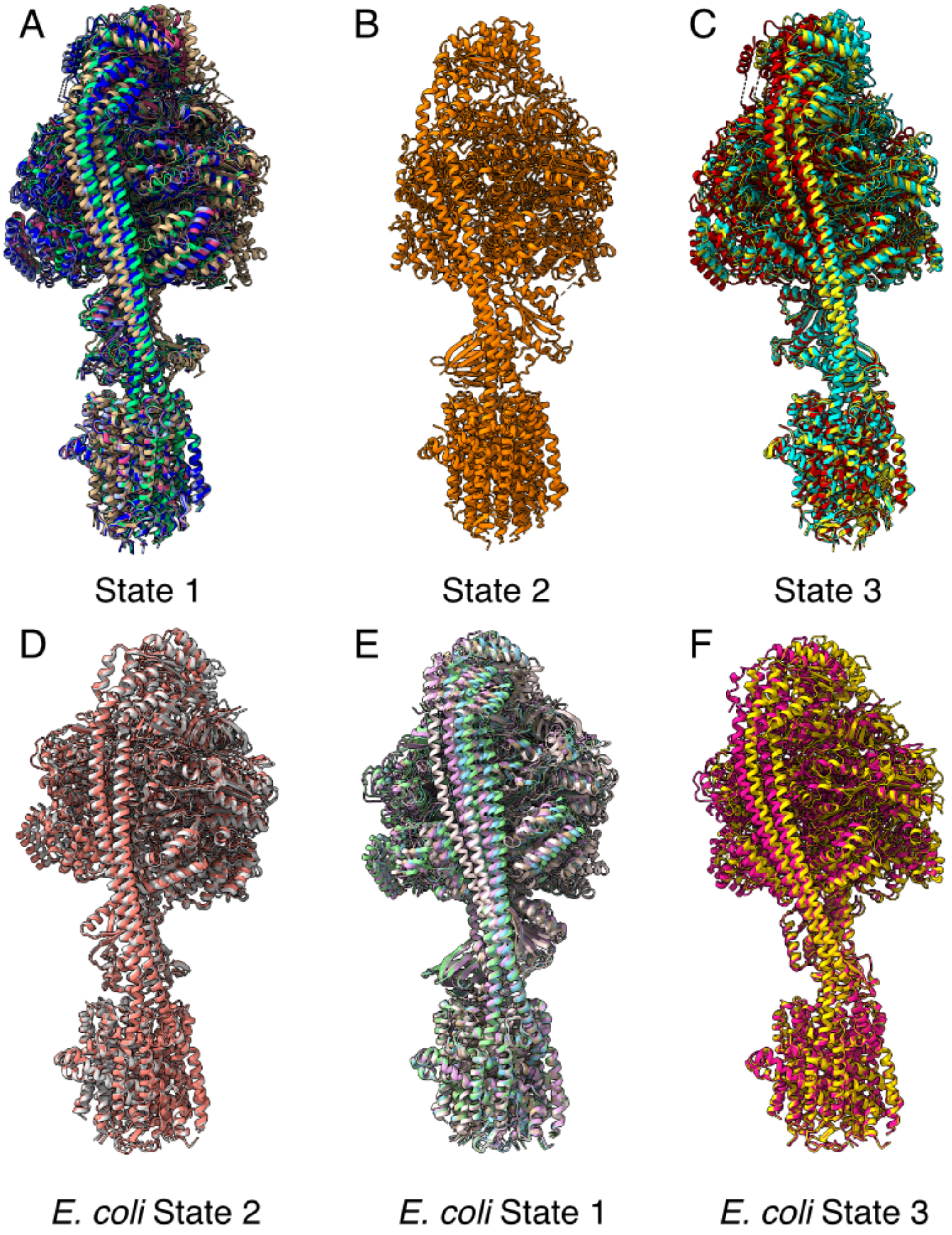

Fig. S11. Views of substates in the ATP synthases from *M. smegmatis* and *E. coli* illustrating differences in the structures of the PS domains. The view is rotated by ~90° relative to Fig. S10. In *E. coli* State1 shows a pronounced lateral movement from left to right. The *M. smegmatis* enzyme appears to move more than the *E. coli* enzyme left to right and in and out of the plane of the page. The figure illustrates that the motions in the PS between species differ, probably because of their different subunit compositions and because the M. smegmatis ε-subunit bulks up the upper part of the rotor.

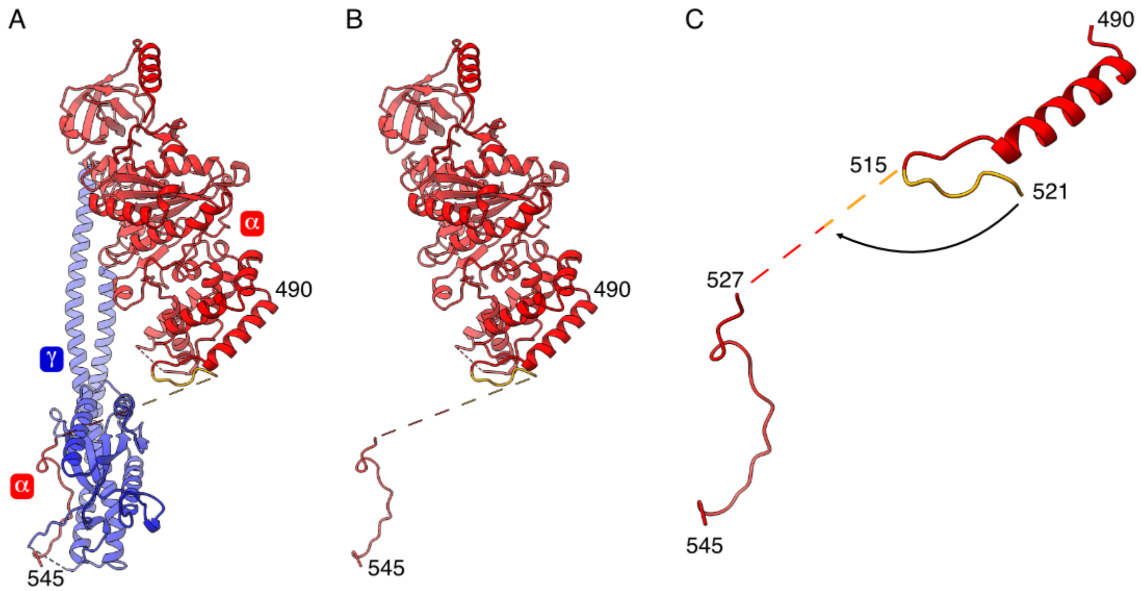

Fig. S12. The inhibitory tether in the ATP synthase from *M. smegmatis* in two conformations. *A*, view of the γ-subunit and an α-subunit illustrating the engagement of the inhibitory tether. *B* and *C*, the extended C-terminal domain of the α-subunit in two conformations. In *B*, the tether extends in the direction of hydrolysis and arrests the rotor via the γ-subunit (residues 527-545 were resolved). In *C*, the tether folds under the α-subunit in the direction of synthesis (residues 515-521 were resolved). During hydrolysis, residues from 521 onwards become caught by the γ-subunit and are pulled in the opposite direction with the tether hinging at around residue 515.

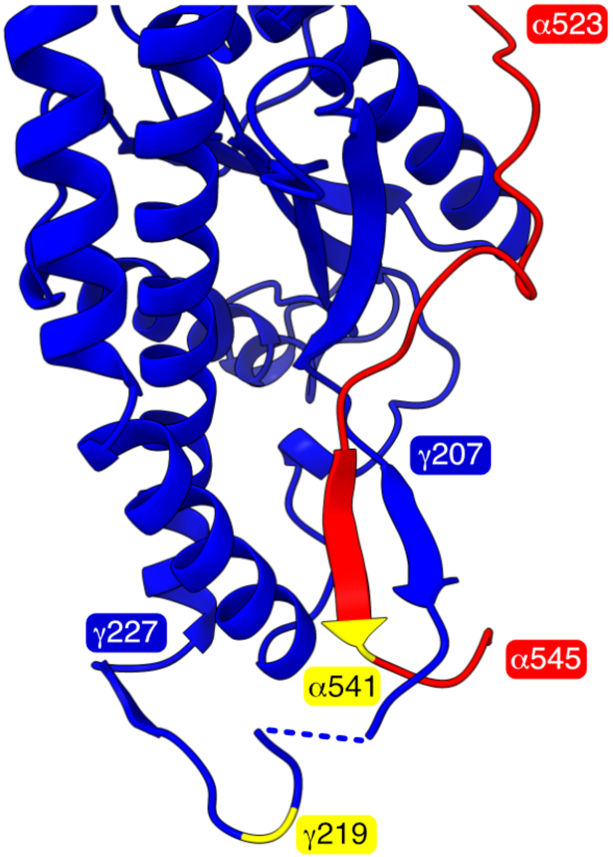

Fig. S13. ***In vitro*** **relief by trypsinolysis of the inhibition of ATP hydrolysis in the ATP synthase from** ***M. smegmatis*****.** Trypsin cleaves the "loop" following γArg219 and the "hook" following αArg541.

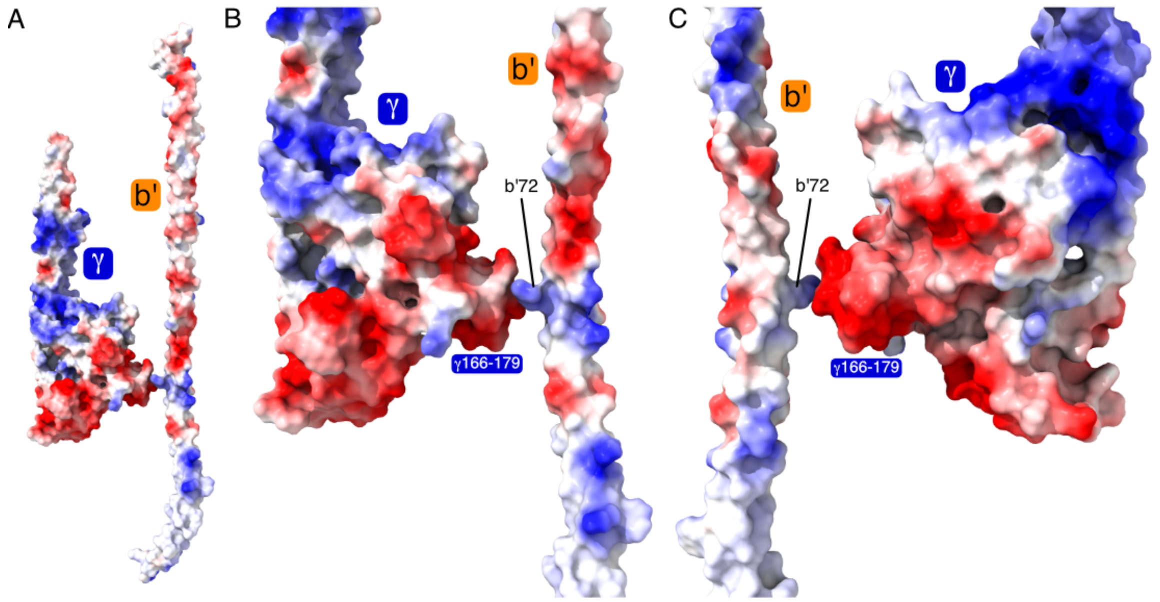

Fig. S14. Electrostatic interactions between the γ-subunit and the PS in the ATP synthase from *M. smegmatis*. The strongly negatively charged residues γ166-179 come very close to the positively charged residue b'Arg72 suggesting the possibility of an interaction. Electrostatic surfaces were calculated in ChimeraX using the s1d model of ATP synthase (PDB 7NJN) where the γ-subunit is closest to the PS. The electrostatic range is red -10, blue +10 V.

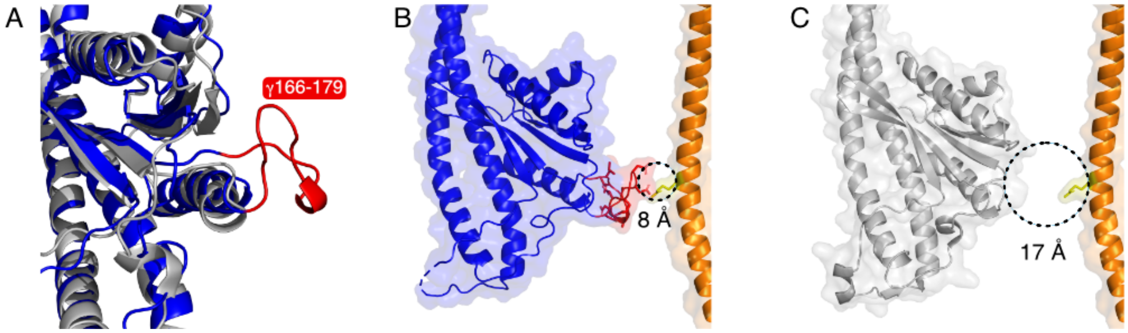

Fig. S15. Comparison of the γ-subunits in the ATP synthases from *M. smegmatis* and *E. coli*. Part (*A)*, the *E. coli* γ-subunit (PDB 6OQV, grey) superimposed on the *M. smegmatis* γ-subunit from s1e shown in blue with residues γ166-179 colored red. Part *(B)*, the *M. smegmatis* γ-subunit and b'-subunit from *M. smegmatis* S1e. The loop formed by residues γ166-179 are unique to mycobacteria and bring the γ-subunit into contact with the PS. Part *(C)*, the same view in the *E. coli* enzyme γ-subunit showing the increased C-α to C-α distance because the *E. coli* γ-subunit lacks the loop residues, and thus there is a significant gap between the γ-subunit and the PS.

```
A0R204|ATPF_MYCS2 MGEFSATILAASQAAEEGGGGSNFLIPNGTFFAVLIIFLIVLGVISKWVVPPISKVLAER 60
P9WPV5|ATPF_MYCTU MGEVSAIVLAASQAAEEGGESSNFLIPNGTFFVVLAIFLVVLAVIGTFVVPPILKVLRER 60
P0ABA0|ATPF_ECOLI -------------------------MNLNATILGQAIAFVLFVLFCMKYVWPPLMAAIEKR 36
6n30  |ATPF_GEOSE ---------------------EAAHGISGGTIIYQLLMFIILLALLRKFAWQPLMNIMKQR 40
                                             ..*::     *::.: .  .:.  *:   : :*

A0R204|ATPF_MYCS2 EAMLAKTAADNRKSAEQVAAAQADYEKEMAEARAQASALRDEARAAG----RSVVDEKRA 116
P9WPV5|ATPF_MYCTU DAMVAKTLADNKKSDEQFAAAQADYDEAMTEARVQASSLRDNARADG----RKVIEDARV 116
P0ABA0|ATPF_ECOLI QKEIADGLASAERAHKDLDLAKASATDQLKKAKAEAQVIIEQANKRR----SQILDEAKA 92
6n30  |ATPF_GEOSE EEHIANEIDQAEKRRQEAEKLLEEQRELMKQSRQEAQALIENARKLAEEQKEQIVASARA 100
                  :  :*.   . .:  ::      .  . : ::: :*. : ::*.        .:: . :.

A0R204|ATPF_MYCS2 QASGEVAQTLTQADQQLSAQGDQVRSGLESSVDGLSAKLASRILGVDVNSGGTQ------ 170
P9WPV5|ATPF_MYCTU RAEQQVASTLQTAHEQLKRERDAVELDLRAHVGTMSATLASRILGVDLTASAATR----- 171
P0ABA0|ATPF_ECOLI EAEQERTKIVAQAQAEIEAERKRAREELRKQVAILAVAGAEKIIERSVDEAANSDIVDKL 152
6n30  |ATPF_GEOSE E----AERVKETAKKEIEREKEQAMAALREQVASLSVLIASKVIEKELTEQDQRKLIEAY 156
                  .           *. ::. : . .   *.  *  ::.  *.:::  .:

A0R204|ATPF_MYCS2 ------------
P9WPV5|ATPF_MYCTU ------------
P0ABA0|ATPF_ECOLI VAEL-------- 156
6n30  |ATPF_GEOSE IKDVQEVGGARM 168
```

Fig. S16. Comparison of sequences of the b'-subunits in *M. smegmatis* (MYCS2), *M. tuberculosis* (MYCTU), *E. coli* and *G. stearothermophilus* (GEOSE). The sequence for *G. stearothermophilus* is taken from PDB 6N30. A suitable match was not found in the UNIPROT database. The sequence of the human b-subunit is substantially different.

```
A0R203|ATPFD_MYCS2 ---------------------------MSIFIGQLIGFAVIAFIIVKWVVPPVRTLMRNQ 33
P9WPV3|ATPFD_MYCTU ---------------------------MSTFIGQLFGFAVIVYLVWRFIVPLVGRLMSAR 33
A0R204|ATPF_MYCS2  MGEFSATILAASQAAEEGGGGSNFLIPNGTFFAVLIIFLIVLGVISKWVVPPISKVLAER 60
P9WPV5|ATPF_MYCTU  MGEVSAIVLAASQAAEEGGESSNFLIPNGTFFVVLAIFLVVLAVIGTFVVPPILKVLRER 60
P0ABA0|ATPF_ECOLI  ------------------------MNLNATILGQAIAFVLFVLFCMKYVWPPLMAAIEKR 36
6n30  |ATPF_GEOSE  --------------------EAAHGISGGTIIYQLLMFIILLALLRKFAWQPLMNIMKQR 40
                                               . ::     * :.  .   :    :   :  :

A0R203|ATPFD_MYCS2 QEAVRAALAESAEAAKKLADADAMHAKALADAKAESEKVTEEAKQDSERIAAQLSE---- 89
P9WPV3|ATPFD_MYCTU QDTVRQQLADAAAAADRLAEASQAHTKALEDAKSEAHRVVEEARTDAERIAEQLEA---- 89
A0R204|ATPF_MYCS2  EAMLAKTAADNRKSAEQVAAAQADYEKEMAEARAQASALRDEARAAG----RSVVDEKRA 116
P9WPV5|ATPF_MYCTU  DAMVAKTLADNKKSDEQFAAAQADYDEAMTEARVQASSLRDNARADG----RKVIEDARV 116
P0ABA0|ATPF_ECOLI  QKEIADGLASAERAHKDLDLAKASATDQLKKAKAEAQVIIEQANKRR----SQILDEAKA 92
6n30  |ATPF_GEOSE  EEHIANEIDQAEKRRQEAEKLLEEQRELMKQSRQEAQALIENARKLAEEQKEQIVASARA 100
                   :  :     .     .          . : .:: ::  : ::*.        .:

A0R203|ATPFD_MYCS2 QAGSEAERIKAQGAQQIQLMRQQLIRQLRTGLGAEAVNKAAEIVRAHVADPQAQSATVDR 149
P9WPV3|ATPFD_MYCTU QADVEAERIKMQGARQVDLIRAQLTRQLRLELGHESVRQARELVRNHVADQAQQSATVDR 149
A0R204|ATPF_MYCS2  QASGEVAQTLTQADQQLSAQGDQVRSGLESSVDGLSAKLASRILGVDVNSGGTQ------ 170
P9WPV5|ATPF_MYCTU  RAEQQVASTLQTAHEQLKRERDAVELDLRAHVGTMSATLASRILGVDLTASAATR----- 171
P0ABA0|ATPF_ECOLI  EAEQERTKIVAQAQAEIEAERKRAREELRKQVAILAVAGAEKIIERSV-DEAANSDIVDK 151
6n30  |ATPF_GEOSE  E----AERVKETAKKEIEREKEQAMAALREQVASLSVLIASKVIEKEL-TEQDQRKLIEA 155
                   .           .  ::.         *.  :   :.  * .::   :

A0R203|ATPFD_MYCS2 FLSELEQM----- 157
P9WPV3|ATPFD_MYCTU FLDQLDAM----- 157
A0R204|ATPF_MYCS2  -------------
P9WPV5|ATPF_MYCTU  -------------
P0ABA0|ATPF_ECOLI  LVAEL-------- 156
6n30  |ATPF_GEOSE  YIKDVQEVGGARM 168
```

Fig. S17. Comparison of sequences of the bδ-subunit and the b'-subunit. Residues 1-157 of the bδ-subunit (ATPFD) are related to the b'-subunits (ATPF) from mycobacteria and other eubacteria. *M. smegmatis* (MYCS2), *M. tuberculosis* (MYCTU), *E. coli* and *G. stearothermophilus* (GEOSE). The sequence for *G. stearothermophilus* is taken from PDB 6N30. A suitable match was not found in the UNIPROT database.

```
A0R203|ATPFD_MYCS2 MSIFIGQLIGFAVIAFIIVKWVVPPVRTLMRNQQEAVRAALAESAEAAKKLADADAMHAK 60
P9WPV3|ATPFD_MYCTU MSTFIGQLFGFAVIVYLVWRFIVPLVGRLMSARQDTVRQQLADAAAAADRLAEASQAHTK 60
P0ABA4|ATPD_ECOLI  ------------------------------------------------------------
6n30  |ATPD_GEOSE  ------------------------------------------------------------
P48047|ATPO_HUMAN  ------------------------------------------------------------

A0R203|ATPFD_MYCS2 ALADAKAESEKVTEEAKQDSERIAAQLSEQAGSEAERIKAQGAQQIQLMRQQLIRQLRTG 120
P9WPV3|ATPFD_MYCTU ALEDAKSEAHRVVEEARTDAERIAEQLEAQADVEAERIKMQGARQVDLIRAQLTRQLRLE 120
P0ABA4|ATPD_ECOLI  ------------------------------------------------------------
6n30  |ATPD_GEOSE  ------------------------------------------------------------
P48047|ATPO_HUMAN  ------------------------------------------------------------

A0R203|ATPFD_MYCS2 LGAEAVNKAAEIVRAHVADPQAQSATVDRFLSELEQMAPSSVVIDTAATSRLRAASRQSL 180
P9WPV3|ATPFD_MYCTU LGHESVRQARELVRNHVADQAQQSATVDRFLDQLDAMAPATADVDYPLLAKMRSASRRAL 180
P0ABA4|ATPD_ECOLI  ------------------------------------------------------------
6n30  |ATPD_GEOSE  ------------------------------------------------------------
P48047|ATPO_HUMAN  ------------------------------------------------------------

A0R203|ATPFD_MYCS2 AALVEKFDSVAGGLDADGLTNLADELASVAKLLLSETALNKHLAEPTDDSAPKVRLLERL 240
P9WPV3|ATPFD_MYCTU TSLVDWFGTMAQDLDHQGLTTLAGELVSVARLLDREAVVTRYLTVPAEDATPRIRLIERL 240
P0ABA4|ATPD_ECOLI  ------------------------------------------------------------
6n30  |ATPD_GEOSE  ------------------------------------------------------------
P48047|ATPO_HUMAN  ------------------------------------------------------------

A0R203|ATPFD_MYCS2 LSDKVSATTLDLLRTAVSNRWSTESNLIDAVEHTARLALLKRAEIAGEVDEVEEQLFRFG 300
P9WPV3|ATPFD_MYCTU VSGKVGAPTLEVLRTAVSKRWSANSDLIDAIEHVSRQALLELAERAGQVDEVEDQLFRFS 300
P0ABA4|ATPD_ECOLI  ------------------------MSEFITVARPYAKAAFDFAVEHQSVERWQDMLAFAA 36
6n30  |ATPD_GEOSE  -------------------------MNQEVIAKRYASALFQIALEQGQLDRIEEDVRAVR 35
P48047|ATPO_HUMAN  ----------------FA-KLVRPPVQVYGIEGRYATALYSAASKQNKLEQVEKELLRVA 43
                                                 :      *  . *    .::. :. :

A0R203|ATPFD_MYCS2 RVLDAEPRLSALLSDYTTPAEGRVALLDKALTGR-PGVNQTAAALLSQTVGLLRGERADE 359
P9WPV3|ATPFD_MYCTU RILDVQPRLAILLGDCAVPAEGRVRLLRKVLERADSTVNPVVVALLSHTVELLRGQAVEE 360
P0ABA4|ATPD_ECOLI  E-VTKNEQMAELLSG----ALAPETLAESFIAVCGEQLDENGQNLIRVMAENGRLNALPD 91
6n30  |ATPD_GEOSE  QALAENGEFLSLL-SYPKLSLDQKKA---LIAEAFAGVSTPVQNTLLLLLERHRFGLVPE 91
P48047|ATPO_HUMAN  QILKEPKVAASVLNPYVKRSIKVKSLNDITAKERFSPL---TTNLINLLAENGRLSNTQG 100
                   . :        :*      :                 :       :       *

A0R203|ATPFD_MYCS2 AVIDLAELAVSRRGEVVAHVSAAAELSDAQRTRLTEVLSRIYGR--PVSVQLHVDPELLG 417
P9WPV3|ATPFD_MYCTU AVLFLAEVAVARRGEIVAQVGAAAELSDAQRTRLTEVLSRIYGH--PVTVQLHIDAALLG 418
P0ABA4|ATPD_ECOLI  VLEQFIHLRAVSEATAEVDVISAAALSEQQLAKISAAMEKRLSR--KVKLNCKIDKSVMA 149
6n30  |ATPD_GEOSE  LAEQFLALVDDARGIAKAVAYSARPLTDEELRALSDVFAQK-VGKQTLEIENIIDPELIG 150
P48047|ATPO_HUMAN  VVSAFSTMMSVHRGEVPCTVTSASPLEEATLSELKTVLKSFLSQGQVLKLEAKTDPSILG 160
                       :  :    ..     . :*  * :     :. .:         : ::   *  ::.

A0R203|ATPFD_MYCS2 GLSITVGDEVIDGSIASRLAAAQTGLPD-- 445
P9WPV3|ATPFD_MYCTU GLSIAVGDEVIDGTLSSRLAAAEARLPD-- 446
P0ABA4|ATPD_ECOLI  GVIIRAGDMVIDGSVRGRLERLADVLQS-- 177
6n30  |ATPD_GEOSE  GVRLRIGNRIYDGSVSGQLERIRRQLIG-- 178
P48047|ATPO_HUMAN  GMIVRIGEKYVDMSVKTKIQKLGRAMREIV 190
                   *: :  *:   * ::  ::      :
```

Fig. S18. Comparison of sequences of the bδ-subunit with the δ-subunit in other species. Residues 264-445 of the bδ-subunit (ATPFD) are related to the δ-subunits (ATPD) from other bacterial species and to the OSCP subunit from mitochondrial ATP synthases. *M. smegmatis* (MYCS2), *M. tuberculosis* (MYCTU), *E. coli* and *G. stearothermophilus* (GEOSE). The sequence for *G. stearothermophilus* is taken from PDB 6N30. A suitable match was not found in the UNIPROT database.

```
bδ 174 ------AASRQSLAALVEKFDSVAGGLDADGLTNLADELASVAKLLLSETALNKHLAEPT 227
bδ 264 ESNLIDAVEHTARLALL-KRAEIAGEVD-----EVEEQLFRFGRVLDAEPRLSALLSDYT 317
             *..: :  **: *  .:** :*     :: ::*  ..::* :*  *.  *:: *

bδ 228 DDSAPKVRLLERLLSDKVS--ATTLDLLRTAVSNRWSTESNLIDAVEHTARLALLKRAEI 285
bδ 318 TPAEGRVALLDKALTGRPGVNQTAAALLSQTVGLLRGERADEAVIDLAELAVSRR----- 372
         :  :* **:: *:.:     *:  **  :*
```

Fig. S19. Comparison of sequences of residues 174-285 and 264-372 of the M. smegmatis bδ-subunit. The bδ-subunit linking sequence appears to have evolved from a duplication of the N-terminal half of the δ-domain. Alignment of linking residues 174-285 with δ-domain residues 264-372 shows reasonable similarity. The regions are structurally similar.

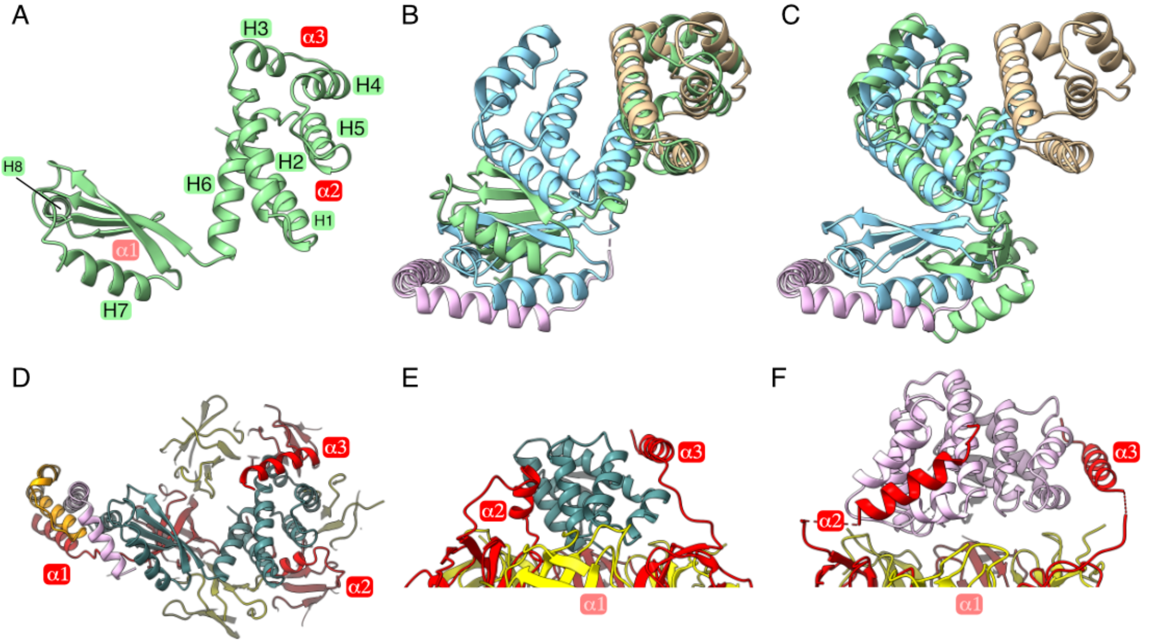

Fig. S20. **Structure of the δ-subunit and its interactions with α-subunits in the ATP synthase from *E. coli*.** *A*, The δ-subunit (green) viewed from above the crown of the F_1-domain towards the IPM consists of eight α-helices, δH1-δH8. The red squares indicate the positions of the three α-subunit N-terminal helices. The N-terminal domain δH1-δH6 shares a structural similarity to the linking domain (bδHb4-δH8) and "δ" domain (bδH10-δH14) of the *M. smegmatis* bδ-subunit. *B*, Superimposition of *E. coli* δ-subunit δH1-δH5 (green) superposed onto the *M. smegmatis* bδ-subunit linking region bδHb4-δH8 (tan). The *M. smegmatis* bδ-subunit "δ" domain and "b" domain are shown in pale blue and pink, respectively. *C*, Similar view to B but a superimposition of *E. coli* δ-subunit δH1-δH5 (green) onto the *M. smegmatis* bδ-subunit "δ" domain bδH10-δH14 (pale blue). *D*, View from above *E. coli* ATP synthase; the δ-, b-, and b'-subunits are colored deep teal, pink and orange respectively and the crown domains of the α- and β-subunits are colored red and yellow, respectively. The interactions of the three α-subunit N-terminal regions are all unique and require these regions to adopt different structures to one another. The structures and interactions of α1 and α3 are similar to those in *M. smegmatis* however, in *E. coli* (and most other species) α2 adopts a shorter helical structure that runs downwards alongside δH1 and δH5 and then under the δ-subunit. *E,* A side view of the *E. coli* enzyme focusing on α2 and α3, and *F*, the same view of the *M. smegmatis* enzyme showing α2 running upwards similar to α3.

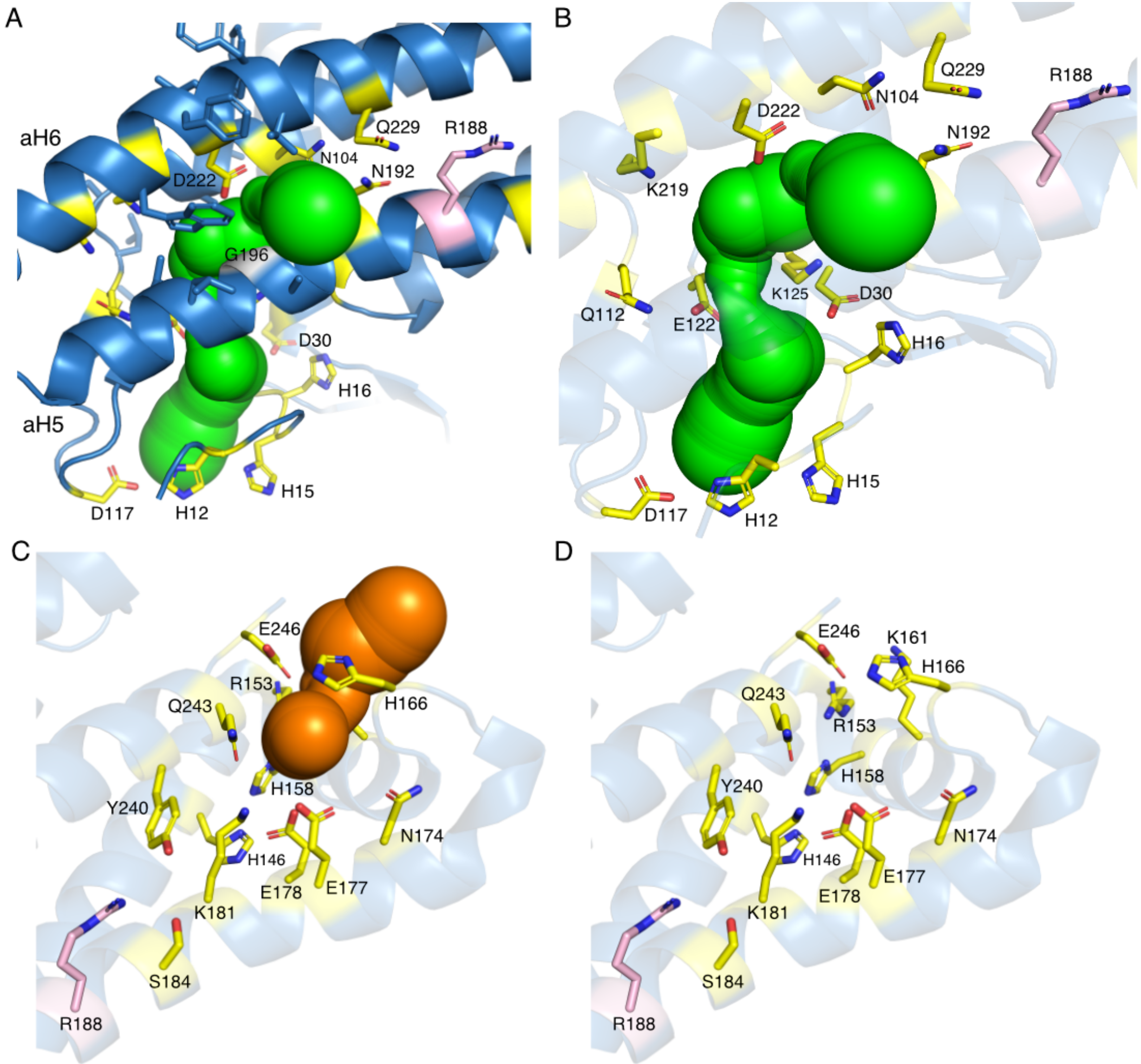

Fig. S21. Details of the inlet and outlet proton channels associated with the *M. smegmatis* a-subunit. The a subunit is predominantly hydrophobic with inlet and outlet channels (green and orange spheres respectively) at the opposite ends of the subunit formed by polar residues. *A-B*, the inlet channel is on the periplasmic underside of the a-subunit near the b-δ subunit. The outlet channel is on the cytosolic upper surface at the opposite end of the a subunit. The inlet channel sits between the two pairs of tilted helices aH3-aH4 and aH5-aH6 and protons are passed (probably via a Grotthuss water chain) through a hole in aH5-aH6 formed by the highly conserved Gly196 residue and polar residue Asp222 (not conserved but always polar). *C-D*, the outlet channel is formed by a series of polar residues leading to a well allowing access to the aqueous cytosol.

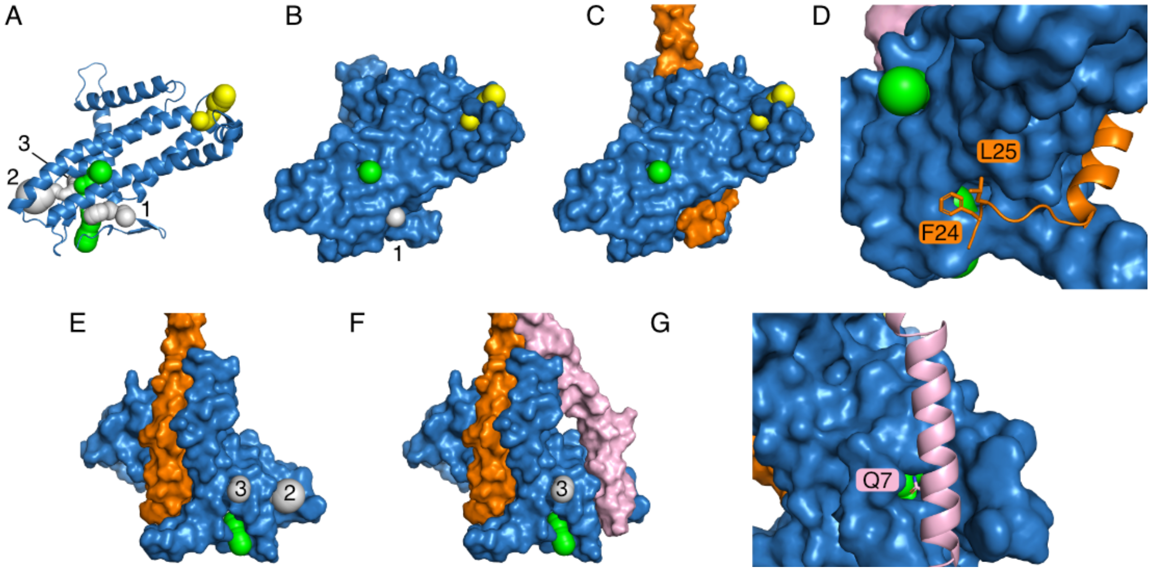

Fig. S22. Additional potential access points to the inlet channel are plugged by subunits bδ and b'. Analysis of the a-subunit using MOLE highlighted three additional points of access to the inlet channel. *A-G*, the a-subunit, shown in sky blue, and the channels found by MOLE shown as spheres. The main inlet channel is shown in green. MOLE found three other channels (shown in grey and numbered 1-3) that lead to the main inlet channel. The outlet channel is shown in yellow. *A-C*, the a-subunit shown in cartoon or surface representation viewed from the c-ring. Channel 1 is on the underside of the a-subunit, has few polar residues nearby and is plugged by the b'-subunit (*C*, shown in orange) via residues F24 and L25. *D*, a closeup view of the residues obscuring the inlet. *E-G*, show the view from the outside (rotated 180° from *A-C*). Channels 2 and 3 enter from the outside, perpendicular to the main inlet channel. Channel 2 is plugged by the bδ-subunit (*F*, shown in pink) via residue Q7 (*G*). Channel 3 has few polar residues nearby and is within the membrane and most likely plugged by lipid. See SI Figure S24 below.

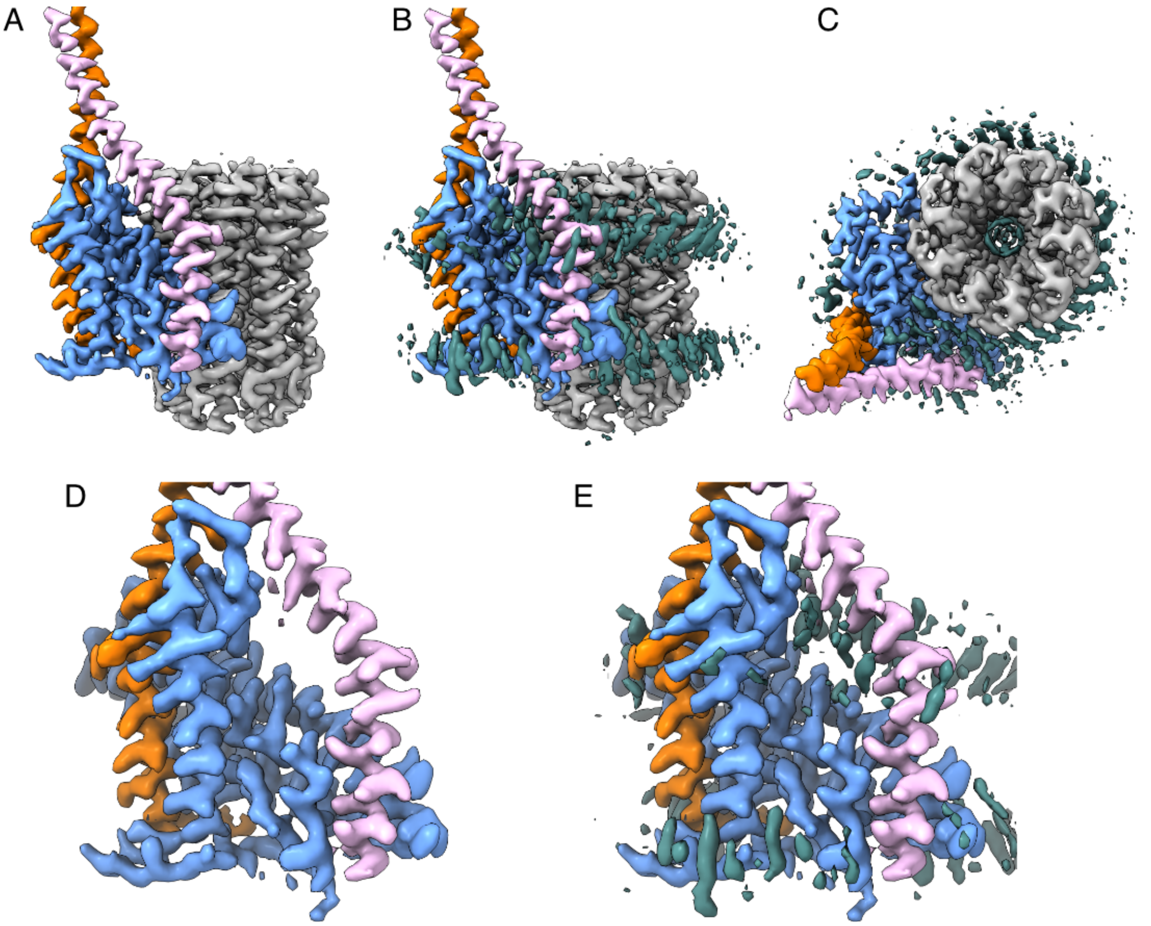

Fig. S23. Arrangement of phospholipids around the a- and bδ-subunits and the c-ring. Phospholipids are arranged around and inside the c-ring and are also found between the a- and bδ-subunits. *A,* side view of the electron density of the a-, bδ-, b'-, and c-subunits. *B,* similar view to *A* but showing the density corresponding to phospholipids/detergent. *C,* view from above showing density within the c-ring. *D,* a closer side view of the the a-, bδ-, and b'-subunits showing a large gap between a and bδ. *E,* the gap is filled with lipid. The electron is colored sky blue, pink, orange, and gray for the a-, bδ-, b'- and c-subunits, respectively. Density corresponding to phospholipids/detergent are shown in teal.

Table S1. Masses of subunits of ATP synthase from *M. smegmatis.*

Subunit	Observed (Da)	Calculated (Da)	Difference (Da)	Possible explanation
α	58,767.0	58,757.6	+9.4	-Met-1
β	51,491.5	51,485.6	+5.9	-Met-1
γ	33,269.4	33,266.7	+2.7	-Met-1
ε	13,131.7	13,133.6	-0.9	-Met-1
a	27,231.8	27,233.3	-1.5	-residues 1-3
bδ	47,473.6	47,449.9	+23.7	+N-formyl
b’	17,613.2	17,620.8	-7.6	
c	8,623.2	8,596.1	+27.1	+N-formyl

Table S2. Summary of the structural model of *M. smegmatis* ATP synthase state 1a.

Subunit	No. of Residues	Residues modelled	Domain	Chain identity
α	548	5-21, 29-406, 411-521	F_1	A
α	548	5-21, 29-406, 411-521	F_1	B
α	548	6-406, 413-521, 527-545	F_1	C
β	475	8-475	F_1	D
β	475	8-471	F_1	E
β	475	7-475	F_1	F
γ	307	3-213, 220-304	F_1	G
ε	121	3-120	F_1	H
a	252	10-247	MD	a
bδ	445	1-162, 169-444	MD, PS	d
b’	170	22-166	MD, PS	b
c	86	3-86	MD	L
c	86	3-86	MD	M
c	86	3-86	MD	N
c	86	3-86	MD	O
c	86	3-86	MD	P
c	86	3-86	MD	Q
c	86	3-86	MD	R
c	86	3-86	MD	S
c	86	3-86	MD	T

Table S3. Deposited data-sets relating to the structure of the ATP synthase from *M. smegmatis*

	EMDB	PDB	Resolution (Å)	B-factor ($Å^2$)	Num. particles	Detail	Comment
**F_1F_o state 1a**	EMD-12377	7NJK	2.52 – 3.40		23,804	Zone composite map	
F_1 state 1a	EMD-12378		2.52	0	"	Local map	
MD s1a	EMD-12379		3.37	-20	"	Local map	
PS s1a	EMD-12380		3.40	-16	"	Local map	
bδ s1a	EMD-12381		3.24	-25	"	Local map	
**F_1F_o state 1b**	EMD-12382	7NJL	2.71 – 3.84		14,078	Zone composite map	
F_1 state 1b	EMD-12383		2.71	-17	"	Local map	
MD state 1b	EMD-12384		3.67	-25	"	Local map	
PS state 1b	EMD-12385		3.77	-18	"	Local map	
bδ state 1b	EMD-12386		3.84	-30	"	Local map	
**F_1F_o state 1c**	EMD-12387	7NJM	2.84 – 3.88		12,277	Zone composite map	
F_1 state 1c	EMD-12388		2.84	0	"	Local map	
MD state 1c	EMD-12389		3.67	-29	"	Local map	
PS state 1c	EMD-12390		3.87	-14	"	Local map	
bδ s1c	EMD-12391		3.73	-30	"	Local map	
**F_1F_o state 1d**	EMD-12392	7NJN	2.64 – 3.99		21,966	Zone composite map	
F_1 state 1d	EMD-12393		2.64	0	"	Local map	
MD state 1d	EMD-12394		3.74	-30	"	Local map	
PS state 1d	EMD-12395		3.99	-10	"	Local map	
bδ state 1d	EMD-12396		3.61	-35	"	Local map	
**F_1F_o state 1e**	EMD-12397	7NJO	2.92 – 4.15		9,602	Zone composite map	
F_1 state 1e	EMD-12398		2.92	0	"	Local map	
MD state 1e	EMD-12399		3.92	-15	"	Local map	
PS state 1e	EMD-12400		4.15	-10	"	Local map	
bδ state 1e	EMD-12401		3.99	-20	"	Local map	
**F_1F_o state 2**	EMD-12402	7NJP	2.84 – 4.07		17,636	Zone composite map	
F_1 state 2	EMD-12403		2.84	-17	"	Local map	
MD state 2	EMD-12404		4.06	-60	"	Local map	
PS state 2	EMD-12405		4.06	-42	"	Local map	
bδ state2	EMD-12406		3.67	-63	"	Local map	
**F_1F_o state 3a**	EMD-12407	7NJQ	2.67 – 3.42		19,516	Zone composite map	
F_1 state 3a	EMD-12408		2.67	0	"	Local map	
MD state 3a	EMD-12409		3.40	-17	"	Local map	

PS state 3a	EMD-12410		3.43	-10	“	Local map	
bδ state 3a	EMD-12411		3.40	-36	“	Local map	
**F_1F_o state 3b**	EMD-12412	7NJR	2.56-3.43		28,438	Zone composite map	
F_1 state 3b	EMD-12413		2.56	0	“	Local map	
MD state 3b	EMD-12414		3.29	-30	“	Local map	
PS state 3b	EMD-12415		3.42	-35	“	Local map	
bδ state 3b	EMD-12416		3.22	-32	“	Local map	
**F_1F_o state 3c**	EMD-12417	7NJS	2.46-3.29		37,894	Zone composite map	
F_1 state 3c	EMD-12418		2.46	0	“	Local map	
MD state 3c	EMD-12419		3.22	-25	“	Local map	
PS state 3c	EMD-12420		3.29	-35	“	Local map	
bδ state 3c	EMD-12421		3.14	-32	“	Local map	
**MD combined All**	EMD-12422	7NJT	2.75	35	184,911		
**MD combined class 1**	EMD-12423	7NJU	3.74	-25	49,823		
**MD combined class 2**	EMD-12424	7NJV	2.90	-25	46961		
**MD combined class 3**	EMD-12425	7NJW	3.67	-25	25,724		
**MD combined class 4**	EMD-12426	7NJX	4.32	-50	16,411		
**MD combined class 5**	EMD-12427	7NJY	2.94	-25	45,992		
**F_1 state 1**	EMD-12432	7NK7	2.11	-15	127,186		
**MD state 1**	EMD-12434	7NK9	2.90	-25	“		
**rotor state 1**	EMD-12436	7NKB	2.77	-25	“		
**bδ state 1**	EMD-12438	7NKD	3.12	-25	“		
**F_1 state 2**	EMD-12439	7NKH	2.78	0	24,938		
**MD state 2**	EMD-12404	7NKP	4.07	-65	17,636	Local map	EMD-12404 above
**rotor state 2**	EMD-12442	7NKK	3.60	-25	24,938		
**bδ state 2**	EMD-12406	7NKL	3.67	-63	17,636	Local map	EMD-12406 above
**F_1 state 3**	EMD-12441	7NKJ	2.17	-15	85,548		
**MD state 3**	EMD-12461	7NL9	2.86	-25	“		
**rotor state 3**	EMD-12445	7NKO	2.71	-25	“		
**bδ state 3**	EMD-12446	7NKQ	2.98	-25	“		

[1] The maps were sharpened with an *ad hoc,* user defined B-factor in lieu of calculating a value from the half-map data.
[2] Without map sharpening.

Table S4. Model refinement statistics

Model	PDB ID	EMDB ID	RMS bonds		Ramachandran		Rotamer outliers (%)	Molprobity score	Clash score	EMRinger score & (cc_mask)
			length (Å)	angles (°)	outliers (%)	favored (%)				
F_1F_o state 1a	7NJK	EMD-12377	0.004	0.519	0.06	97.58	0.0	1.32	4.52	4.35 (0.87)
F_1F_o state 1b	7NJL	EMD-12382	0.003	0.511	0.04	97.59	0.0	1.43	6.23	3.77 (0.86)
F_1F_o state 1c	7NJM	EMD-12387	0.003	0.500	0.04	97.29	0.0	1.49	6.44	3.49 (0.86)
F_1F_o state 1d	7NJN	EMD-12392	0.004	0.520	0.06	97.60	0.0	1.42	6.08	3.94 (0.87)
F_1F_o state 1e	7NJO	EMD-12397	0.004	0.534	0.04	97.07	0.0	1.60	8.06	3.17 (0.85)
F_1F_o state 2	7NJP	EMD-12402	0.003	0.499	0.04	97.70	0.0	1.41	6.27	3.44 (0.86)
F_1F_o state 3a	7NJQ	EMD-12407	0.004	0.523	0.06	97.81	0.0	1.33	5.38	4.10 (0.87)
F_1F_o state 3b	7NJR	EMD-12412	0.004	0.522	0.06	96.92	0.0	1.43	4.85	4.22 (0.87)
F_1F_o state 3c	7NJS	EMD-12417	0.002	0.500	0.02	98.30	1.22	1.25	3.98	4.05 (0.88)
MD combined all	7NJT	EMD-12422	0.003	0.482	0.00	97.27	0.0	1.44	5.52	2.39 (0.87)
MD combined class 1	7NJU	EMD-12423	0.003	0.468	0.00	95.98	0.0	1.58	5.54	1.57 (0.83)
MD combined class 2	7NJV	EMD-12424	0.003	0.472	0.00	97.81	0.0	1.35	5.58	2.74 (0.88)
MD combined class 3	7NJW	EMD-12425	0.003	0.504	0.00	96.80	0.0	1.61	7.49	1.69 (0.82)
MD combined class 4	7NJX	EMD-12426	0.003	0.519	0.00	94.24	0.0	1.99	12.24	0.54 (0.76)
MD combined class 5	7NJY	EMD-12427	0.003	0.471	0.00	97.17	0.0	1.39	4.69	2.98 (0.87)

F_1 state 1	7NK7	EMD-12432	0.006	0.632	0.07	98.13	0.88	1.12	3.31	5.91 (0.89)
MD state 1	7NK9	EMD-12434	0.003	0.495	0.00	97.14	0.0	1.51	6.42	1.78 (0.86)
rotor state 1	7NKB	EMD-12436	0.004	0.505	0.0	98.04	0.0	1.36	6.56	2.90 (0.86)
bδ state 1	7NKD	EMD-12438	0.003	0.452	0.0	98.29	0.0	1.36	6.49	2.57 (0.81)
F_1 state 2	7NKH	EMD-12439	0.002	0.506	0.07	97.82	0.00	1.21	3.79	4.82 (0.88)
MD state 2	7NKP	EMD-12404	0.004	0.531	0.00	96.08	0.0	1.89	13.04	0.82 (0.77)
rotor state 2	7NKK	EMD-12442	0.003	0.512	0.0	96.69	0.0	1.76	10.68	1.78 (0.80)
bδ state 2	7NKL	EMD-12406	0.003	0.491	0.0	97.62	0.0	1.50	7.56	1.86 (0.77)
F_1 state 3	7NKJ	EMD-12441	0.005	0.592	0.07	98.15	1.05	1.07	2.66	6.04 (0.89)
MD state 3	7NL9	EMD-12461	0.003	0.475	0.0	97.48	0.0	1.44	6.02	2.20 (0.86)
rotor state 3	7NKN	EMD-12444	0.003	0.473	0.0	97.43	0.0	1.36	4.81	3.48 (0.86)
bδ state 3	7NKQ	EMD-12446	0.004	0.490	0.0	97.28	0.0	1.52	6.97	2.98 (0.81)

Movie legends

Movie S1 (separate file). The cryo-em structure of the ATP synthase from *M. smegmatis*. The composite cryo-em density map (EMB-12377) of the ATP synthase from *M. smegmatis*, in catalytic State1a, is shown and rotated through 360°. The resolution of the composite map is 2.52 – 3.40 Å. The regions of density corresponding to the α-, β-, γ-, ε-, a-, bδ-, b'-, and c-subunits are colored red, yellow, blue, green, sky blue, pink, orange, and grey, respectively.
FILE: Movie-S1_s1a_360_map_15fps_super3_945_180fr.mp4

Movie S2 (separate file). Movements of the ATP synthase from *M. smegmatis* during ATP synthesis. An animation showing an interpolation of the ATP synthase substate models in the direction of synthesis created using the morph function in ChimeraX. The order of the procession of substates shown is S1a>S1b>S1e>S1d>S1c>S2>S3c>S3a>S3b. Residues 527-545 of the α-subunits have been omitted to avoid misrepresentation of interpolation errors in the auto-inhibitory tether. Subunits are colored according to Fig. 1 and the atomic model is shown in a space-fill representation,
FILE: Movie-S2_MsF1Fo_s1abedc_s2_s3cab_spacePSr_syncol_15fps_super3.mp4

Movie S3 (separate file). Alternative interpolation of movements of the ATP synthase from *M. smegmatis* during ATP synthesis. An animation showing an interpolation of the ATP synthase substate models in the direction of synthesis created using the morph function in ChimeraX. An alternative order of the State 3 substates is presented. Here, the order of the procession of substates shown is S1a>S1b>S1e>S1d>S1c>S2>S3b>S3a>S3c. Residues 527-545 of the α-subunits have been omitted to avoid misrepresentation of interpolation errors in the auto-inhibitory tether. Subunits are colored according to Fig. 1 and the atomic model is shown in a space-fill representation,
FILE: Movie-S3_MsF1Fo_s1abedc_s2_s3bac_spacePSr_syncol_15fps_super3.mp4

Movie S4 (separate file). Procession of the c_9-ring during ATP synthesis. A view from above, (the cytoplasmic side of the periplasmic membrane), showing the a-, bδ-, and b-subunits and the c-ring. The c-subunit monomer located directly under the ε-subunit is colored red. As above in Movies S2 and S3, the substate models have been interpolated to show the movement of the c_9-ring and, by extension, the rotor during synthesis. The order of the procession of substates shown is S1a>S1b>S1e>S1d>S1c>S2>S3c>S3a>S3b. Whilst there is an alternate order for the State3 substates presented for the whole complex (Movie S2), the position of the c-ring in both interpretations is the same.
FILE: Ms_states_ring_s1abedc23cab_grey_15fps_super3.mp4

Movie S5 (separate file). Movements of the ATP synthase from *M. smegmatis* during ATP hydrolysis (spacefill representation). An animation showing an interpolation of the ATP synthase substate models in the direction of hydrolysis created using the morph function in ChimeraX. The order of the procession of substates shown is S1c>S1e>S1d>S1b>S1a>S3b>S3a>S3c>S2. Movements of α-subunit residues 527-545, which comprise the auto-inhibitory "tether", should be taken as indicative rather than factual. In circumstances where the tether residues are bound to the γ-subunit rotation of the enzyme cannot proceed as shown and, whilst the trajectory of the extended tether region toward its association with the "loop" to form the auto-inhibited complex can be speculated, the data presented here describe only its final position rather than its movement.
FILE: Movie-S5_MsF1Fo_s1cedba_s3bac_s2_spacePSr_hydcol_15fps_super3.mp4

Movie S6 (separate file). Detail of the auto-inhibitory tether bound to the γ-subunit of the ATP synthase from *M. smegmatis* in State S3. A view of the electron density and fitted atomic model of the lower portion of the enzyme's rotor in catalytic State3 showing the binding mode of the auto-inhibitory "tether" of the α-subunit. The γ-, ε-, and α- subunits are colored blue, green and red, respectively, and the c_9-ring, at bottom of the screen, is shown in grey. The view is then rotated through 360 degrees. As demonstrated, the C-terminal residues of the α-subunit form the "hook" of the tether which locks into the catching "loop" provided by residues 212-220 of the γ-subunit.

Once formed, this association sterically prohibits rotation during hydrolysis, stalling the rotary action, to inhibit continued hydrolysis by the enzyme.
FILE: Movie-S6_rotor_s3_360_cart_15fps_super3_180fr_700b.mp4

Movie S7 (separate file). Structural duplication in the b-δ domain maintains common interaction interfaces with the α-subunits. The movie illustrates that the bδH4-bδH8 linking domain of the mycobacterial δ-subunit can be superimposed onto the δ-domain by a clockwise rotation of *ca.*120°, and that the two domains each interact with the N-terminal region of two separate α-subunits in similar ways.
FILE: Movie-S7_b-delta_duplication_alphas_10fps_super3wait.mp4

REFERENCES

1. G. Bashiri, A.M. Rehan, D.R. Greenwood, J.M. Dickson, E.N. Baker, Metabolic engineering of cofactor F420 production in Mycobacterium smegmatis. *PLoS One* **5**, e15803 (2010).
2. J.R. Meyerson *et al.*, Self-assembled monolayers improve protein distribution on holey carbon cryo-EM supports. *Sci Rep* **4**, 7084 (2014).
3. J. Zivanov *et al.*, New tools for automated high-resolution cryo-EM structure determination in RELION-3. *Elife* **7**, (2018).
4. A. Rohou, N. Grigorieff, CTFFIND4: Fast and accurate defocus estimation from electron micrographs. *J Struct Biol* **192**, 216-221 (2015).
5. T.D. Goddard *et al.*, UCSF ChimeraX: Meeting modern challenges in visualization and analysis. *Protein Sci* **27**, 14-25 (2018).
6. S. Chen *et al.*, High-resolution noise substitution to measure overfitting and validate resolution in 3D structure determination by single particle electron cryomicroscopy. *Ultramicroscopy* **135**, 24-35 (2013).
7. P. Emsley, B. Lohkamp, W.G. Scott, K. Cowtan, Features and development of Coot. *Acta Crystallogr D Biol Crystallogr* **66**, 486-501 (2010).
8. P.V. Afonine *et al.*, Real-space refinement in PHENIX for cryo-EM and crystallography. *Acta Crystallogr D Struct Biol* **74**, 531-544 (2018).
9. A.T. Zhang, M.G. Montgomery, A.G.W. Leslie, G.M. Cook, J.E. Walker, The structure of the catalytic domain of the ATP synthase from Mycobacterium smegmatis is a target for developing antitubercular drugs. *Proc Natl Acad Sci U S A* **116**, 4206-4211 (2019).
10. L. Preiss *et al.*, Structure of the mycobacterial ATP synthase Fo rotor ring in complex with the anti-TB drug bedaquiline. *Sci Adv* **1**, e1500106 (2015).
11. D. Liebschner *et al.*, Macromolecular structure determination using X-rays, neutrons and electrons: recent developments in Phenix. *Acta Crystallogr D Struct Biol* **75**, 861-877 (2019).
12. V.B. Chen *et al.*, MolProbity: all-atom structure validation for macromolecular crystallography. *Acta Crystallogr D Biol Crystallogr* **66**, 12-21 (2010).
13. B.A. Barad *et al.*, EMRinger: side chain-directed model and map validation for 3D cryo-electron microscopy. *Nat Methods* **12**, 943-946 (2015).
14. K. Berka *et al.*, MOLEonline 2.0: interactive web-based analysis of biomacromolecular channels. *Nucleic Acids Res* **40**, W222-7 (2012).
15. Schrodinger, LLC, The PyMOL Molecular Graphics System, Version 2.4.2. (2018).
16. H. Guo *et al.*, Structure of mycobacterial ATP synthase bound to the tuberculosis drug bedaquiline. *Nature* **589**, 143-147 (2021).
